# Biopsy vs. extensive resection for first recurrence of glioblastoma: is a prospective clinical trial warranted?

**DOI:** 10.1186/s13104-015-1386-3

**Published:** 2015-09-04

**Authors:** Christopher Dardis, Lynn Ashby, William Shapiro, Nader Sanai

**Affiliations:** Department of Neurology, Barrow Neurological Institute, Suite 300, 500 West Thomas Road, Phoenix, AZ 85013 USA; Department of Neurosurgery, Barrow Neurological Institute, Phoenix, AZ 85013 USA

**Keywords:** Glioblastoma, Recurrent, Progressive, Resection, Biopsy, Sample size

## Abstract

**Background:**

Glioblastoma is an aggressive and almost universally fatal tumor. The prognosis at the time of recurrence has generally been poor, with overall survival typically in the range of 4–40 weeks. The merits of surgical resection (vs. open biopsy, to confirm recurrence via histology) in addition to conventional adjuvant chemotherapy have been the subject of longstanding debate. We wondered whether it would possible to conduct a trial at our institution to settle this question definitively with Class I evidence.

**Results:**

Initially, we had hoped to conduct a randomized, unblinded prospective clinical trial. However on closer inspection it appeared that such an undertaking would pose significant practical challenges. Thus we present our protocol in draft form. In keeping with recommended outcomes for these tumors, the primary endpoint would be median progression free survival. Secondary end points would be: median overall survival (mOS, from time of recurrence) and change in Karnofsky Performance Status over time. Patients would be eligible at the time of first recurrence if they had received conventional treatment until that point and at least 1 month had elapsed since the time of radiation. All patients would be considered potentially eligible for enrollment (unless the decision regarding resection was already clear-cut in view of other factors). Using Cox’s proportional hazards model, we estimate that at least 456 patients would be necessary to demonstrate an increase in the hazard ratio to 1.3 for those undergoing biopsy alone. This magnitude of benefit is estimated based on a review of retrospective studies.

**Discussion:**

If restricted to our Institution alone, which sees approximately 100–150 new cases of glioblastoma each year, a trial of this nature would be likely to take around 10 years. Furthermore, there may be significant reluctance on the part of patients and physicians to participate. There is also the opportunity cost of excluding patients from other trials to consider. We recognize that the estimate of the magnitude of effect may be conservative. As things stand, we feel that multi-institutional collaboration would almost certainly be required for an undertaking of this kind.

**Electronic supplementary material:**

The online version of this article (doi:10.1186/s13104-015-1386-3) contains supplementary material, which is available to authorized users.

## Background

Glioblastoma (GB), meaning World Health Organization Grade IV astrocytoma, is the most common primary brain tumor in adults [1]. These tumors are almost universally fatal. Its annual incidence is estimated to be 3.2/100,000 in the United States [2]. Standard therapy for newly diagnosed patients with GB involves maximal surgical resection, followed by radiotherapy (RT, typically 60 Gray, given in 30 fractions) with concomitant and adjuvant temozolomide (TMZ) for at least 6 months. The addition of TMZ to RT has increased median overall survival (mOS) from 12.1 months to 14.6 months, and 2-year survival from 10 to 26 % [3].

Recurrence of GB is almost inevitable. A meta-analysis of 8 Phase II studies involving 225 patients with recurrent GB showed a mOS after disease recurrence of just 25 weeks and a progression-free survival rate at 6 months (PFS-6) of only 15 % [4].

Accurately diagnosing recurrence remains a clinical challenge. The standard adjuvant treatments of RT and TMZ lead to pseudo-progression on follow-up imaging in 20–30 % of patients imaged at 2 months [5]. In order to differentiate tumor recurrence from radiographic pseudo-progression, a surgical specimen for histological analysis remains the reference standard.

### Role of surgery at recurrence

In a minority of cases surgery is essential in order to relieve mass effect caused by tumor growth. However, once recurrence of tumor has been confirmed, surgery often proceeds with the goal of removal of as much of the remaining tumor as possible. This is particularly likely to be the case when the diagnosis can be determined during surgery via frozen section.

There has been considerable debate about the merits of such a strategy. This is reflected, for example, in the Canadian recommendations for the treatment of recurrent or progressive GB, which state: “In the absence of level I evidence, the decision to re-operate should be made according to individual circumstances, in consultation with the multidisciplinary team and the patient” [6]. By contrast, the USA’s National Comprehensive Cancer Network guideline for recurrent (local) GB favors resection when possible [7].

Where facilities are available, a consensus has developed that resection is advisable if the patient is well enough and the tumor accessible. It has been proposed that approximately 25 % of patients are eligible for repeat resection at recurrence or progression [8].

Historically, the earliest published work on the question, from 1967, favors surgical resection for recurrent glioma (including GB). The authors view is that surgery requires as radical a removal of tumor as is feasible [9]. With some refinements, this has remained the general consensus since.

Clearly, *some* form of treatment is generally advisable at recurrence. This was argued in 2003 when a strategy of aggressive treatment [surgery, RT and chemotherapy (CT)] compared favorably with no intervention (n = 90 vs. n = 78). Regarding surgery, the authors state that: “the major criteria guiding this decision were location and tumor size (both influencing the chance of a macroscopic total resection), mass effect, and the impact of surgery on additional strategies” [10].

Other important retrospective series addressing the question appear in Table 1 [11–16]. This represents a spectrum of opinion. Some strongly favor re-operation [17–19]. Others have been more sanguine [20, 21].

**Table 1:** Estimates of survival following recurrence of glioblastoma

	N	Tx	mPFS	mOS
Series involving resection ± additional adjuvant Tx at recurrence				
Quick [26]	29	GRT ± RT, CT	NA	66.5
	11	SRT ± RT, CT	NA	37.4
DeBonis [11]	17	Sx	NA	26 (13–39)
	19	No Tx	NA	22 (13–30)
	24	CT	NA	35 (22–43)
	16	Sx + CT ± RT	NA	61 (43–78)
Clarke [24]	758	CT (P II)	8.3 (8.0–9.6)	31.4 (25.3–35.9)
Park [27]	34	Sx	NA	4–46.8 varies by NRGS
McGirt [20]	294	Sx (GTR)	NA	51
Mandl [14]	9	Sx (≥1)	NA	13
	11	Sx + SRS/CT	NA	34
	12	SRS/CT	NA	28
Hau [10]	90	Sx, RT, CT	13 (10–13)	33 (26–39)
	78	No Tx	15 % at 12 mos	9 (4–15)
Pinsker [13]	38		NA	18–21
Guyotat [20]	18	Sx	NA	22.6
	36	No Sx	NA	8.6
Barker [21]	43	Sx (≥1)	NA	42 (37–50)
	130	No Sx	NA	23 (20–29)
Landy [11]	12	Sx ± RT, CT	NA	8
Harsh [17]	39	Sx	10 with KPS >70	36
Ammirati [18]	35	Sx ± RT, CT	NA	29
Series using adjuvant bevacizumab at recurrence				
Friedman [31]	85	Bev	18.5 (12.6–25.2)	40 (35.6–46.5)
Kresyl [32]	48	Bev	16 (12–26)	31 (21–54)
Series using CT (not bevacizumab) at recurrence				
Lamborn [33]	437	CT (P II)	8 (8–9)	30 (27–33)
Wong [4]	225	CT (P II)	9 (8–10)	25 (21–28)

Brackets indicate 95 % CI where available

*n* number of patients in study, *Tx* treatment, *Sx* surgery, *RT* radiotherapy, *CT* chemotherapy, *mPFS* median progression free survival (weeks), *mOS* median overall survival (weeks), *KPS* Karnofsky performance status, *NA* not available, *LRCT* locoregional CT (i.e. bleomycin + mitoxantrone via Ommaya reservoir), *P II* Phase II studies

The role of resection at recurrence of GB (including *second* recurrence and beyond), has most forcefully been argued by Chaichana et al. in 2013 in their cohort of 579 patients [22]. The authors conclude that: “patients who underwent an increasing number of resections had increased survival benefit regardless of age, functional status, and other factors”. They do acknowledge the caveat that their findings “may be limited by an inherent bias in patient selection, which may favor patients with more benign tumor biology”.

Nonetheless, the most comprehensive review on the question of resection to date (evaluating 11 studies) concluded that there is *no* established role for surgery in this setting [23]. The patient’s age and Karnofsky Performance Status (KPS) were identified as important prognostic predictors and, to a lesser extent, the size of the tumor.

The largest single retrospective study to address the question has been that of the North American Brain Tumor Consortium. The group looked at Phase II studies in recurrent GB (1998–2005, n = 511) and concluded that progression-free survival and overall survival are similar irrespective of surgery. [24] They suggest that surgery at recurrence “balances the scales, permitting patients who would otherwise do worse due to bulky tumor to do as well as patients who do not require surgery”.

Other recent reviews have tended to be more favorable to resection. One from 2008 concluded that reoperation probably provides an average improvement of 3–5 months in mOS. Amongst the conclusions are that the ‘ideal’ patient is “less than 50 years old (although older patients can benefit), has a KPS of greater than or equal to 60–70; [has a] tumor in a favorable location; [has a tumor where] a 98 % resection is possible; and [has been progression free for] more than 6 months since the initial diagnosis” [25].

Another, from 2014, with 40 patients, tended to favor surgery and concluded that patients in good clinical condition should not have the option of second surgery withheld [26].

Stereotactic radiosurgery (SRS), particularly Gamma Knife^®^, has also been proposed as a safe and effective alternative to a second resection in selected patients [27]. Similarly to our own proposal regarding resection, a review of studies to date (nine studies, n = 283 cases) addressing the value of radiosurgery in this setting also favors a randomized clinical trial (RCT) to address the question of selection bias [28].

### Stratification

Scoring systems have been proposed to help individualize decision making in these circumstances. The first of these, in 2010, the NIH Recurrent GB Scale (NRGS), is a 0–3 point scale which identified the following as independent predictors of poor prognosis:

- postoperative KPS score ≤80
- tumor volume ≥50 cm^3^
- involvement of ≥2 of 3 critical areas of cortex:
  - Motor
  - Speech
  - MCA (areas adjacent to the M1/M2 areas of the Middle Cerebral Artery)

This was derived from (n = 34) and validated with (n = 109) a cohort of patients who all underwent surgery. The authors propose that those with ≤2 points “have significantly longer expected postoperative survival periods, and [that] reoperation, if indicated, should be pursued” [29].

Another series from 2013 identified two important predictors of poor survival: [30]

- KPS ≤70
- enhancement of the ventricular wall

Again, this was derived from (n = 55) and validated with (n = 96) patients undergoing surgery. The authors recommend that “for patients with a score of 2 [i.e. having both risk factors], surgery is not recommended and conservative management may be better”.

### Role of bevacizumab at recurrence

Regardless of the decision on whether to proceed with surgery, some form of additional CT at recurrence is generally agreed to be worthwhile where possible. Although no one agent has yet been endorsed by existing guidelines, all of them give prime consideration of bevacizumab. Although no Phase III trial has been performed to validate this strategy, it has been approved by the Federal and Drug Administration (FDA) on the basis of who Phase II studies, both published in 2009. In that by Friedman et al., those using bevacizumab alone had a PFS-6 of 43 % and mOS of 40 weeks (95 % CI 35.6–46.5) [31]. That by Kresyl et al. showed a PFS-6 of 57 % (95 % CI 44–75) and mOS of 31 weeks (95 % CI 21–54) [32]. This was a favorable outcome with respect to historical controls (those treated with temozolomide alone).

At least one trial undertaken since then has called into question the use of single-agent bevacizumab as adjuvant chemotherapy when GB progresses. However a consensus has yet to emerge on whether dual-agent treatment should be implemented routinely in this setting. [33]

## Results

### Trial design

**Fig. 1:**
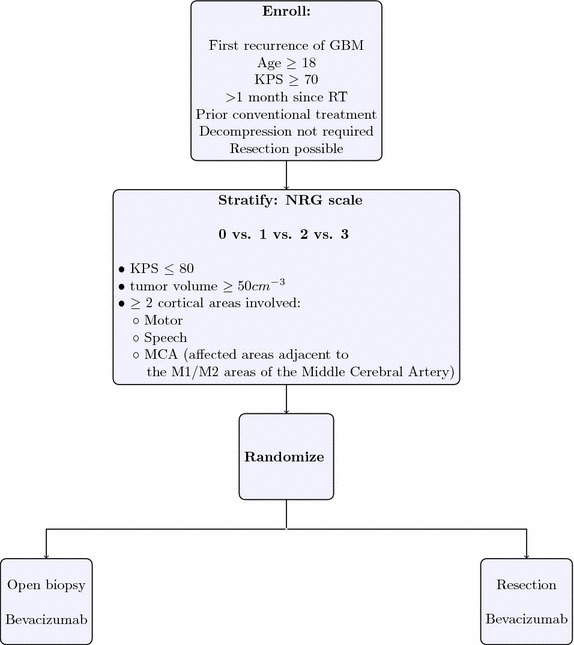
Trial design overview

The protocol itself is available as a Additional file 1. The document was generated using LaTeX. Those wishing to adapt the protocol can also find a printout of the source code used to generate this as Additional file 2. The sections which follow refer to this draft protocol, which is presented as a single-institution study. An overview of the trial design is presented in Fig. 1.

Based on the retrospective data in Table 1, we believe that extensive resection is likely to improve mPFS. Owing to the lack of prospective data to guide the decision on the extent of resection at recurrence, we designed an RCT (unblinded) to settle the question more definitively. The goal is to generate Class I evidence to guide clinicians in this difficult decision.

### Endpoints

The primary endpoint is mPFS after first recurrence of GB [34]. Secondary endpoints are: PFS-6 after first recurrence of GB, mOS and change in KPS over time. Progression is to be determined by clinical judgment, supplemented by imaging. As KPS is an ordinal scale, we propose to compare KPS scores at 3 and 6 months using non-parametric tests.

If there was robust evidence in favor of resection, we also plan to perform regression to attempt to control for additional variables which are known to be associated with outcome. These include the NRGS as above as well as age, KPS and proportion of tumor resected. This latter would be calculated from the volume on enhancement on pre- and post- operative MRIs. However, as the trial sample size is based on the effect size of the intervention on the primary endpoint and as the trial stops at interim analysis if this endpoint is reached, it is likely to be under-powered to control adequately for these confounders. As the extent of resection cannot be determined accurately prior to surgery, there is no easy way to control for this through stratified randomization.

### Initial treatment

Initial treatment is according to our current standard of care. Patients with suspicion of GB based on clinical and radiological characteristics would undergo surgery; if the frozen section confirmed the diagnosis, an attempt at maximal resection would be pursued.

Following this, intensity-modulated radiation treatment (IMRT) is given (typically 55–60 Gy) with concurrent and adjuvant TMZ as per the original Stupp protocol [35]. This starts within 8 weeks of the primary resection (typically 3 weeks). Adjuvant TMZ is administered at cycles 5 days/month for at least 6 months. Although the Stupp protocol continued adjuvant treatment for 6 months, it has since become accepted practice to continue this for at least 12 months. Beyond 12 months, the use of TMZ is individualized. We plan to continue treatment beyond this as tolerated, based on a retrospective series showing signs of benefit [36].

We accept patients whose initial care had taken place elsewhere, provided their care has been similar to that outlined above, as judged on an intention-to-treat basis.

### Surgery at recurrence

Patients are considered eligible for surgery at recurrence based on the opinion of their treating Neurosurgeon. All adult patients with a KPS of ≥70 are considered potentially eligible at the time of recurrence, unless there are factors present which would ‘tip the scales’ entirely in one direction; for example major systemic co-morbidity, tumor in an unresectable location (bi-thalamic, diffuse pontine) or mass effect from the tumor putting the patient at immediate risk of complications such as herniation or blockage of a major artery.

Patients randomized to resection undergo routine, image-guided cyto-reduction targeting the contrast-enhancing portion of the lesion on T1-weighted contrast-enhanced magnetic resonance imaging (MRI). Intraoperative adjuncts, including, and not limited to, 5-aminolevulinic acid, intraoperative MRI and intraoperative monitoring using electrodes, are included as needed per the treating Neurosurgeon. Carmustine (Gliadel^®^) wafers are not implanted.

Patients randomized to open biopsy undergo an image-guided biopsy using a minimal craniotomy and open microsurgical techniques (This differs from a stereotactic needle biopsy. We prefer an open biopsy due to its superior diagnostic accuracy [37]).

### Further chemotherapy

Treatment is with bevacizumab, 10 mg/kg every 2 weeks. The first dose is given 2–4 weeks after the date of surgery or biopsy. This is continued indefinitely (as tolerated) until time of progression. Full details of reporting and management of adverse events are included in the protocol.

### Monitoring

Patients follow up with their Neuro-Oncologist at monthly intervals. MRIs are repeated every 2 months, or sooner if new symptoms or signs occur which are concerning for disease progression. KPS is assessed at each of these visits.

### Treatment at time of progression

At time of subsequent progression, the patient is re-evaluated at our weekly Multidisciplinary Central Nervous System Tumor Conference. Decisions are individualized but include the options of further surgery, further IMRT, SRS, a change in chemotherapy, treatment with electrical fields (Optune^®^) and Palliative Care.

### Sample size considerations

Following a review of cases seen at our Institution from 2009 to 2012, we estimate 50–70 cases/year of first recurrence of GB. We estimate that 60 % will be eligible for entry to and agreeable to participation in the study. We realize this figure is higher than the 25 % previously cited but felt this more realistically represents current practice in our setting [8].

Following standard statistical methods, we assume that the survival distributions for both groups will be exponential [38]. The hazard radio (HR) is thus the reciprocal of the ratio of median survival times. For example if the mPFS was 17 weeks in those treated with biopsy and 34 weeks with surgery, the HR is 1/(17/34) = 2. Based on Table 1 as well as our own experience, we believe that the mPFS in those treated with surgery will be closer to 23 weeks, giving a HR of approximately 1.3 (or 1/1.3 = 77 % for patients undergoing resection vs. biopsy alone).

The numbers required for various effect sizes and values for the power of the test are illustrated in Table 2. These are derived, as is typical, using the method of Schoenfeld, where all subjects are followed until the event of interest occurs (progression or death). [39] Significance is two-sided, meaning that those undergoing biopsy only have 1.4× the risk of progression in the period of time under consideration. Thus with a HR of 1.3 and a power of 80 %, the trial would require 456 participants and would need to run for 11 years (40 entrants/year). This assumes that *all* patients proceed with the intervention to which they are randomized and that all are followed until death or further progression of disease. For GB it appears a reasonable assumption that that all will progress again at some point.

**Table 2:** Sample size necessary to demonstrate a significant difference in hazard ratio (HR)

Power (%)	HR		
	1.2	1.3	2.0
70	742	359	51
80	945	456	65
90	1264	611	87.5

Assumes two-sided significance (alpha) of 5 %

### Costing

While precise costing is beyond the scope of the present paper, these are likely to be small relative to oncologic trials in general. In particular, both resection and open biopsy are considered ‘standard of care’, as are all of the specified treatments. Thus the only costs would be administrative: help from a Nurse trained in clinical research would be valuable in helping to explain the consent form to patients and their families; help would also be valuable for data entry and adverse event reporting, given the constraints on the treating physicians’ time. Additional administrative costs may arise from a data-monitoring committee and Institutional Review Board. Bearing all of this in mind, we feel that a trial such as this could be run for less than $60,000 per year.

## Discussion

### Similar trials

Ours in not a novel design. We are aware of another a similar RCT designed to compare the use of bevacizumab with or without surgery at time of progression of GB [40]. This study was withdrawn from ‘clinicaltrials.gov’ before patient enrollment began. The sample size is substantially smaller, with 42 participants allocated to each arm; thus the estimated effect size is expected to be much larger; for a sample size of 84, the HR would need to be approximately 1.75 to demonstrate a difference via the log-rank test (assuming complete follow up). The primarily endpoint, mOS differs from ours. Also, patients are not required to have histologically confirmed recurrence at the time of progression, as in our protocol.

### Estimates of effect and sample size

We acknowledge that our estimate of the HR may be overly cautious. Likewise, our service is growing over time so that our estimate of the number of eligible patients each year may be too small. As explained below, we feel it is worth erring on the side of caution in the design of a trial such as this.

The difficulties caused by insufficient sample size in surgical trials have been highlighted in diverse fields; for example, reviews of the topic have been undertaken in Orthopedic and Cardiothoracic Surgery [41, 42]. The authors of the latter review conclude that “for most study questions in clinical surgery, comparative analysis of large case series and databases will provide more robust evidence”. We will return to this suggestion in the conclusions.

Estimating the magnitude of effect size in a trial such as this is made more challenging owing to the heterogeneity of reported outcomes in the existing literature. Over time, a consensus has emerged that mPFS is the likely to be the most valuable measure of efficacy in recurrent GB. The older studies have tended to favor mOS as an endpoint. However this is becoming complicated by an increasing range of therapeutic options when signs of further progression develop. Furthermore, interventions tend to become increasingly individualized as the disease progresses. These later interventions would be challenging to standardize and may obscure the earlier effects of initial treatment on outcome.

**Fig. 2:**
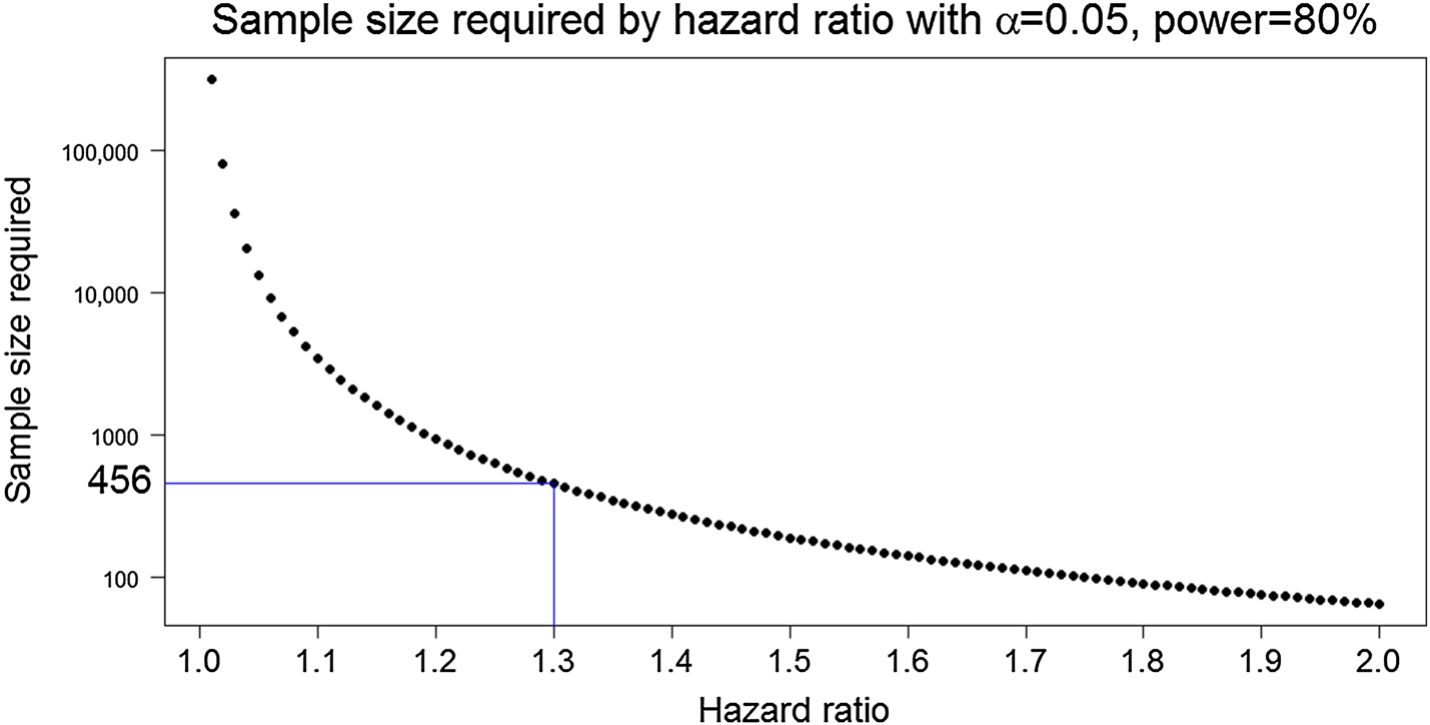
Graph showing the relationship between required hazard ratio and sample size. Sample size is plotted on a log scale (base 10). Significance (alpha, two-sided) = 0.05. Power = 80 %

While we settled on a HR of 1.3, which would lead to a sample size of at least 456, a more conservative design would allow for a sample size sufficient to demonstrate a difference between groups with a HR of 1.2. This would more than double the sample size, to 945, which is large by the standards of clinical trials in this field. The relation of sample size to HR is not a linear and as the HR approaches 1 the sample size increases dramatically. This is illustrated in Fig. 2. This figure of 456 is something of a ‘tipping point’ in that larger sizes appear beyond the scope of most centers acting individually. A trial such as this would, ideally, only need to be performed once. This more conservative estimate would mitigate against the trial being under-powered and inconclusive. If an annual review of results to date were to be performed according to the traditional methods of O’Brien and Fleming, it would still have the potential to end long before the target sample size had accrued [43].

While such a small improvement (HR of 1.2) may seem overly pessimistic, it would at least avoid the difficulty of interpreting a negative result with a smaller number of patients enrolled [44]. The corollary is that there is little to lose by *over*-*powering* this type of trial; indeed once the required organizational structure was in place, it could readily be adapted to similar questions in the field.

### A multi-institutional endeavor?

Such an undertaking is likely to prove difficult for any single center and thus multi-institutional collaboration, possibly international, is likely to be necessary. Such an approach has already proved feasible in other trials of adjuvant treatments for GB, with target sample sizes of over 950 now achievable [45]. Given that there are an estimated 18,000 patients newly diagnosed with GB each year in the USA alone, then with sufficient co-ordination even a sample size such as this could accrue rapidly.

In a multi-institutional trial of surgery there would be a great need to ensure standardization of techniques. The variability due to different treatment centers can be accounted for using ‘clustered’ analysis, or multilevel/'mixed-effects’ models, although such approaches may further inflate the required sample size. There may also be resistance from local Neurosurgeons to the imposition of standardization and to randomization taking place remotely.

### Treatment preferences: patient and physician

Bias, in the form of patient preference, appears to be the biggest obstacle to the design of randomized trials in surgery. It is likely that there exists an inherent, intuitive bias in favor of surgery in most patients. This appears to be particularly true for the evaluation of treatments for malignancy, in the comparison or surgical with non-surgical approaches and where survival is a primary endpoint [46]. *All* of these factors are present to some degree in our draft protocol.

Bias may also exist for the Neurosurgeon. In order to randomize the decision to proceed with surgery at the time of recurrence, true clinical equipoise is required. We believe this is unlikely to be the case in the majority of our patients, when considered from the perspective of the treating Neurosurgeon. By proposing randomization, the treating clinicians may feel that their judgment and experience are being undermined. In addition, institutions seeing patients with this condition are likely to have established preferences for attempt at resection vs. biopsy. Difficulties such as this have been identified as a barrier to recruitment in other clinical trials and feelings of lack of equipoise appear to be greater among surgeons than other physicians [47, 48].

### Alternatives to the RCT

To overcome these difficulties, a number of alternatives to the traditional RCT have been proposed [49]. One possibility is ‘pre-randomization’, whereby randomization occurs *prior* to informed consent i.e. the patient is presented with the recommended treatment without consideration of an alternative. While attractive theoretically, we suggest that in the modern regulatory context this is unlikely to pass muster with most Institutional Review Boards.

Another is the ‘patient preference’ design; this allows the treating patient and surgeon to decide on appropriate treatment; only where lack of preference exists is randomization performed. This mirrors the ‘real-world’ scenario most closely. One difficulty here is that the arms are likely to become weighted towards one treatment, making statistical conclusions fraught. Furthermore, this design is likely to have an imbalance in confounding variables such that the results would be less likely to gain acceptance.

In any of these designs, including the RCT, is has been suggested that the traditional *p* < 0.05 criteria for significance be relaxed to take account of the difficulties inherent in surgical trials. This appears justified, in that *smaller* numbers of subjects will tend to *increase* the *p* value (unless the null hypothesis is true). However, no clear consensus exists as to what alternative level of significance to use.

The simplest approach to a question such as this is a historical-control trial (HCT). This has been discouraged on the grounds that HCTs tend to have more positive outcomes that those following a RCT design. The most through review on the subject, involving six common medical conditions for which 50 RCTs and 56 HCTs were available, showed that the proposed treatment improved outcomes in 80 % of the HCTs and only 20 % of the RCTs. This difference was attributed to differences in the control group and remained after HCTs were adjusted for confounding prognostic factors [50]. The same authors give a sensitivity and specificity for these methods, shown in Table 3 [51]. Admittedly, only one of these interventions was surgical (surgery for esophageal varices).

**Table 3:** Sensitivity and specificity of randomized controlled and historical control trials

Method	Sensitivity	Specificity
Randomized controlled	0.12 (0–0.27)	0.88 (0.67–1.0)
Historical controls	0.9 (0.8–1)	0.11 (0–0.27)

Figures are given as mean (range) [40]

### Effect on other trials

There would be an ‘opportunity-cost’ of participating in our protocol. Patients would lose the opportunity to participate in other studies. There are typically a large number of such trials enrolling for the treatment of first recurrence of GB, the majority of which involve CT (at the time of writing there were approximately 20 in the category of immunotherapy alone) [52]. Many of these will have stronger expectations of benefit than the advantages we would attribute to second resection (vs. open biopsy). This is particularly the case in that the disease is likely to have become more multifocal and diffuse at the time of recurrence, even if this pattern is not obvious on conventional MRI. Thus, more systemically-targeted treatments are likely to be stronger candidates for consideration at the time of disease progression.

## Conclusions

Even with a modest improvement in outcome, we feel that such a trial would be worthwhile. However, while attractive on theoretical grounds, a trial such as this is faced with a number of barriers to practical implementation. The greatest amongst these are the sample size required and the probable duration of the trial. These are far from insurmountable.

For now, it is likely that we will have to settle for the technique of use of historical controls. Despite the shortcomings of this approach, we hold to the maxim that ‘the good is not the enemy of the best’. In due course, the cumulative evidence from reported cases should establish a consensus. With greater standardization of stratifying predictors and of outcomes, and with sufficient pooling of data, this should become increasingly tractable.

## Additional files

**Additional file 1.** Draft protocol for a trial of Biopsy vs. Extensive Resection for recurrent Glioblastoma (the BERG trial).
**Additional file 2.** LaTeX code used to generate the BERG trial protocol. This is provided so that others can modify the protocol or use elements from it.

## Abbreviations

GB: glioblastoma
TMZ: temozolomide
RT: radiotherapy
CT: chemotherapy
mPFS: median progression free survival
mOS: median overall survival
PFS-6: progression-free survival at 6 months
KPS: Karnofsky Performance Score
NRGS: NIH Recurrent GBM Scale (NIH = National Institute of Health)
FDA: Food and Drug Administration
IMRT: intensity-modulated radiation treatment
MRI: magnetic resonance imaging
SRS: stereotactic radiosurgery
HR: hazard ratio
RCT: randomized controlled trial
HCT: historical-control trial
