# Supplementary material for: Biopsy vs. extensive resection for first recurrence of glioblastoma: is a prospective clinical trial warranted?
Source: BMC Res Notes. 2015 Sep 4;8:414. doi: 10.1186/s13104-015-1386-3 (PMC4560929; doi:10.1186/s13104-015-1386-3)
Supplement: Supplementary file 1 — Additional file 1. Draft protocol for a trial of Biopsy vs. Extensive Resection for recurrent Glioblastoma (the BERG trial). [file 13104_2015_1386_MOESM1_ESM.pdf]

Biopsy vs Extensive Resection for first recurrence of Glioblastoma: The **BERG** trail

Christopher Dardis (CD)

Version 0.2 from 7/01/2014

Revision History

**Revision**	**Date**	**Author(s)**	**Description**
0.1	10/18/2013	CD	Created
0.2	7/01/2014	CD	Updated

Contents

1 Study team 3

2 Schema 4

Abbreviations 5

3 Eligibility 7

4 Registration 9

5 Rationale 11

6 Objectives 12

7 Surgery 12

8 Drug treatment: bevacizumab 13

9 Monitoring 23

10 Treatment at time of progression 23

11 Statistical considerations 23

12 Data collection 26

A Sample consent form 28

B Rights of humans subjects in medical experiments 33

C Karnofsky performance scale 34

1 Study team

1.1 Principal Investigator (Neurosurgery)

Nunc sed pede. Praesent vitae lectus. Praesent neque justo, vehicula eget, interdum id, facilisis et, nibh. Phasellus at purus et libero lacinia dictum. Fusce aliquet. Nulla eu ante placerat leo semper dictum. Mauris metus. Curabitur lobortis. Curabitur sollicitudin hendrerit nunc. Donec ultrices lacus id ipsum.

1.2 Co-Investigator (Neuro-Oncology)

Nunc sed pede. Praesent vitae lectus. Praesent neque justo, vehicula eget, interdum id, facilisis et, nibh. Phasellus at purus et libero lacinia dictum. Fusce aliquet. Nulla eu ante placerat leo semper dictum. Mauris metus. Curabitur lobortis. Curabitur sollicitudin hendrerit nunc. Donec ultrices lacus id ipsum.

1.3 Neurosurgery:

Pellentesque interdum sapien sed nulla. Proin tincidunt. Aliquam volutpat est vel massa. Sed dolor lacus, imperdiet non, ornare non, commodo eu, neque. Integer pretium semper justo. Proin risus. Nullam id quam. Nam neque. Duis vitae wisi ullamcorper diam congue ultricies. Quisque ligula. Mauris vehicula.

1.4 Neuro-Oncology:

Pellentesque interdum sapien sed nulla. Proin tincidunt. Aliquam volutpat est vel massa. Sed dolor lacus, imperdiet non, ornare non, commodo eu, neque. Integer pretium semper justo. Proin risus. Nullam id quam. Nam neque. Duis vitae wisi ullamcorper diam congue ultricies. Quisque ligula. Mauris vehicula.

1.5 Radiation Oncology:

Pellentesque interdum sapien sed nulla. Proin tincidunt. Aliquam volutpat est vel massa. Sed dolor lacus, imperdiet non, ornare non, commodo eu, neque. Integer pretium semper justo. Proin risus. Nullam id quam. Nam neque. Duis vitae wisi ullamcorper diam congue ultricies. Quisque ligula. Mauris vehicula.

2 Schema

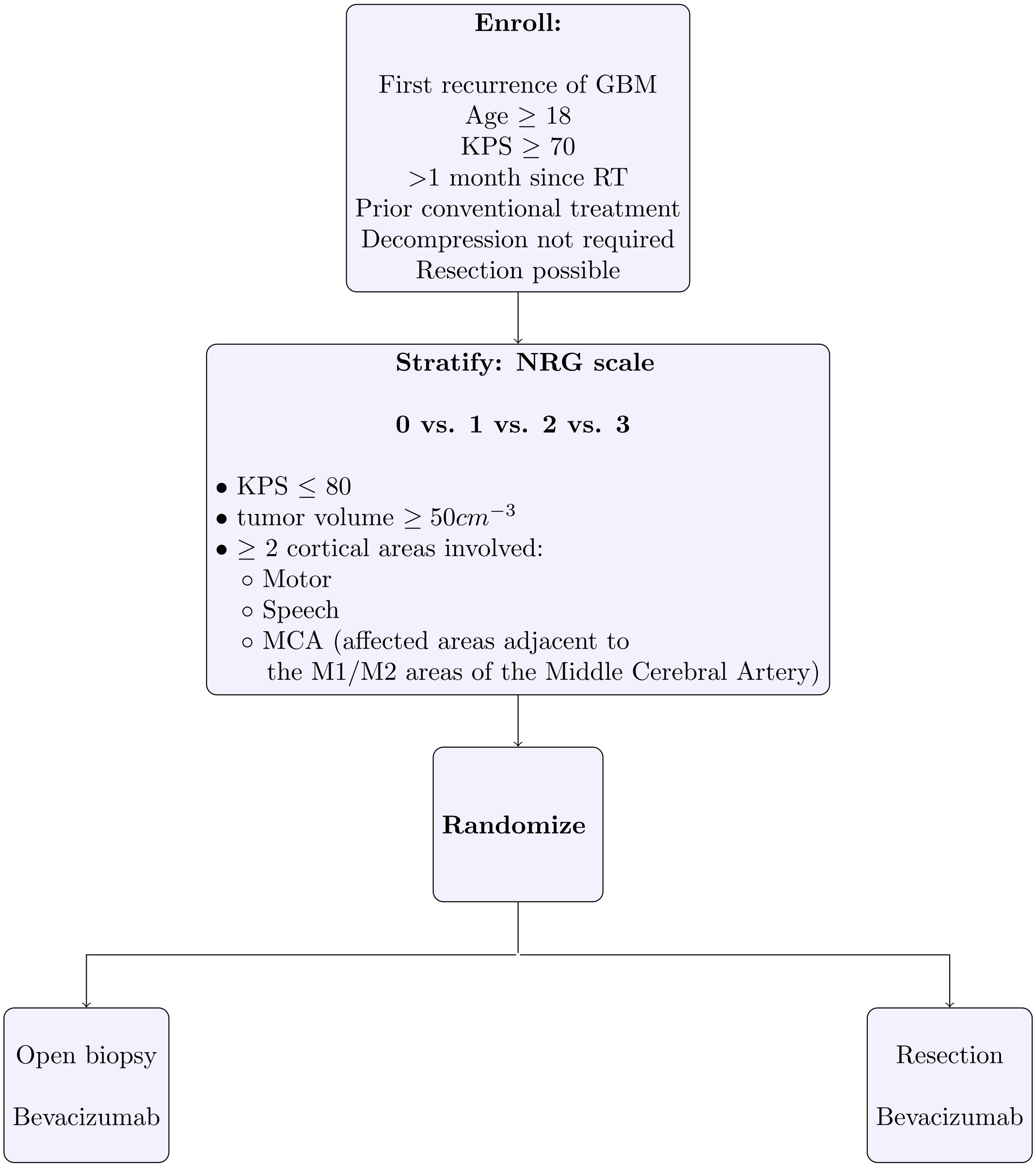
Enroll:
First recurrence of GBM
Age ≥ 18
KPS ≥ 70
>1 month since RT
Prior conventional treatment
Decompression not required
Resection possible
Stratify: NRG scale
0 vs. 1 vs. 2 vs. 3
• KPS ≤ 80
• tumor volume $\geq 50cm^{-3}$
• $\geq$ 2 cortical areas involved:
◦ Motor
◦ Speech
◦ MCA (affected areas adjacent to the M1/M2 areas of the Middle Cerebral Artery)
Randomize
Open biopsy
Bevacizumab
Resection
Bevacizumab

Abbreviations

AEs adverse events. 13

ALT alanine aminotransferase. 6, 15

ANC absolute neutrophil count. 6, 15

AST aspartate transaminase. 6, 15

bHCG beta human chorionic gonadotropin. 7

BP blood pressure. 6

BSA body surface area. 8

CAEPR Comprehensive Adverse Event and Potential Risks list. 13

CNS central nervous system. 19, 21, 24

CT computer automated tomography. 6

CTEP-AERS Cancer Therapy Evaluation Program Adverse Event Reporting System. 13

DHHS Department of Health and Human Services. 29

eGFR estimated glomerular filtration rate (MDRD method). 6

FDA Food and Drug Administration. 11, 25, 27, 29

FSH follicle-stimulating hormone. 16

GBM glioblastoma multiforme. 6, 10, 11, 23

Hb hemoglobin. 6

HR hazard ratio. 22, 23

INR international normalized ratio. 6

IRB institutional review board. 25

IV intravenous. 12, 16, 28

KPS Karnofsky performance status. 6, 8, 10, 11, 22, 24

mAb monoclonal antibody. 12

mOS median overall survival. 10, 11, 23, 24

MRI magnetic resonance imaging. 6, 11, 21, 22

NCI National Cancer Institute. 13

PFS-6 progression free survival at 6 months. 10, 11, 23, 24

PRES posterior reversible leukoencephalopathy syndrome. 21

RPLS reversible posterior leukoencephalopathy syndrome. 16, 21

RT radiation therapy. 6, 10, 22, 23

RTOG Radiation Therapy Oncology Group. 13

SPEER Specific Protocol Exceptions to Expedited Reporting. 13

TMZ temozolomide. 10, 23

ULN upper limit of normal (defined for institution). 6

UPC urine protein creatinine. 20

VEGF vascular endothelial growth factor. 12

WCC white cell count. 6, 15

WNL within normal limits. 20

3 Eligibility

Eligibility checklist

1. - ☐ History of glioblastoma multiforme (GBM)
2. - ☐ First recurrence
3. - ☐ Age >18
4. - ☐ Karnofsky performance status (KPS) $\geq$ 70
5. - ☐ >1 month since radiation therapy (RT)
6. - ☐ Prior conventional treatment (Stupp regimen)
7. - ☐ **No** need for surgical decompression
8. - ☐ Resection possible
9. - ☐ **No** sign diffuse spread of disease or leptomeningeal disease
10. 14 days prior to enrollment:
    (a) - ☐ History & physical exam
    (b) - ☐ Brain imaging shows probable signs of progression of disease. magnetic resonance imaging (MRI) scans are preferred but computer automated tomography (CT) is acceptable if there is a contraindication to the use of MRI
    (c) - ☐ Steroid dose stable or decreasing for at least 7 days
    (d) - ☐ Blood pressure (BP) - systolic <160, diastolic <90. Use of antihypertensives permitted
    (e) - ☐ White cell count (WCC) >3,000/mm^3
    (f) - ☐ Absolute neutrophil count (ANC) >1,500/mm^3
    (g) - ☐ Platelets >100,000/mm^3
    (h) - ☐ Hemoglobin (Hb) >10 g/dLt
    (i) - ☐ Aspartate transaminase (AST), alanine aminotransferase (ALT), bilirubin <3 upper limit of normal (defined for institution) (ULN)
    (j) - ☐ International normalized ratio (INR) <1.5 ULN
    (k) - ☐ Estimated glomerular filtration rate (MDRD method) (eGFR) >60mL/min/1.73m^2

(l) - ☐ If potentially pregnant - serum beta human chorionic gonadotropin (bHCG) negative

11. - ☐ 1 month prior: **no** major surgery

12. - ☐ 3 months prior: **no** history of non-healing wounds, ulcers or bone fractures

13. 6 months prior:

    (a) - ☐ **No** unstable angina or myocardial infarct

    (b) - ☐ **No** heart failure requiring hospitalization

    (c) - ☐ **No** stroke or transient ischemic attack

14. - ☐ 3 years prior: **No** other malignancy (not including completely excised basal or squamous cell skin cancer)

15. - ☐ If sexually active, agrees to practice adequate contraception during study and for 6 months thereafter

16. - ☐ **No** major autoimmune condition requiring immune suppression e.g. etanercept, infliximab, ivIg

17. - ☐ **No** prior treatment with anti-VEGF targeted treatments e.g. bevacizumab, cediranib, vandetanib, aflibercept, sunitinib, sorafenib

The Eligibility Checklist must be completed in its entirety prior to registration. The completed, signed, and dated checklist used at study entry will be retained in the patients study file.

4 Registration

4.1 Procedure

Patient registration will be conducted in accordance with routine hospital protocol. Enrolled patients will be further registered in a de-identified database maintained by the Research Nurse.

4.2 Data collection at registration

Checklist for study enrollment

1. - ☐ Name of person randomizing case
2. - ☐ Eligibility checklist completed
3. - ☐ Investigator considers patient eligible
4. Informed consent:
   (a) - ☐ For trial participation
   (b) - ☐ For tissue and blood to be kept for use in research to learn about, prevent or treat cancer
   (c) - ☐ To allow someone from this institution to contact him or her in the future to take part in more research
5. Patient:
   (a) - ☐ Initials
   (b) - ☐ Identification number
   (c) - ☐ Gender
   (d) - ☐ Date of birth
   (e) - ☐ KPS
   (f) - ☐ Weight
   (g) - ☐ Body surface area (BSA)
6. Physicians:
   (a) - ☐ Neurosurgeon
   (b) - ☐ Oncologist (Neuro-Oncology or Medical Oncology)
   (c) - ☐ Radiation Oncologist

7. Dates:

   (a) - ☐ First resection

   (b) - ☐ Started radiation

   (c) - ☐ Completed radiation

   (d) - ☐ Started temozolomide

   (e) - ☐ Completed temozolomide

   (f) - ☐ Registered for this trial

   (g) - ☐ Randomized for this trial

____

Printed name

____

Completed by

____

Date

5 Rationale

5.1 Background

GBM (World Health Organization Grade IV astrocytoma), is the most common primary brain tumor in adults and portends a very poor prognosis. [1] Its incidence is estimated to be 3.2/100,000 in the United States. [2]

Standard therapy for patients with newly diagnosed GBM usually involves maximal surgical resection, followed by RT (typically 60 Gray, given in 30 fractions) with concomitant and adjuvant temozolomide (TMZ) for at least 6 months. The addition of TMZ to RT has increased median overall survival (mOS) from 12.1 months to 14.6 months, and 2-year survival from 10% to 26%. [4]

Recurrence of GBM is almost inevitable. A meta-analysis of 8 Phase II studies involving 225 patients with recurrent GBM, mOS after disease recurrence was 25 weeks and the progression free survival at 6 months (PFS-6) was 15%. [5]

Accurately diagnosing recurrence remains a challenge. Pseudo-progression occurs on follow-up imaging in 20-30% of patients imaged at 2 months when treated with standard adjuvant RT and TMZ. [3]. In order to differentiate tumor recurrence from radiographic pseudo-progression, a surgical specimen remains the gold standard.

5.2 Surgery

In some cases surgery is essential in order to relieve mass effect caused by tumor growth. However in most cases, once recurrence of tumor has been confirmed (typically by frozen-section performed at the time of surgery), surgery proceeds with the goal of removal of as much of the remaining tumor as possible.

There has been considerable debate about the merits of such a strategy. This is reflected, for example, in the Canadian recommendations for the treatment of recurrent or progressive GBM, which states

> In the absence of level 1 evidence, the decision to re-operate should be made according to individual circumstances, in consultation with the multidisciplinary team and the patient.

[6]

By contrast the National Comprehensive Cancer Network guideline for recurrent (local) GBM favors resection when possible. [7]

A recent review of literature on the subject, the most comprehensive to date (evaluating 11 studies), concluded that there is no established role for surgery in this setting. [8] Age and KPS were identified as generally important prognostic predictors and to a lesser extent, size of tumor.

5.3 Bevacizumab

Regardless of the decision on whether to proceed with surgery (of whatever extent), some form of additional chemotherapy at recurrence is generally agreed to be worthwhile where possible. Although no one agent has yet been endorsed by existing guidelines, all of them give prime consideration of bevacizumab.

Although no phase III trial has been performed to validate this strategy, it has been approved by the Food and Drug Administration (FDA) on the basis of two phase II studies, both published in 2009. In that by Friedman et al., those using bevacizumab alone had a PFS-6 of 43% and mOS of 40 (95% CI 35.6-46.5) weeks. [9] That by Kresyl et al. showed a mOS of 31 (95%CI 21-54) weeks. PFS-6 was 57% (95%CI 44-75). [12] This was a favorable outcome with respect to historical controls, for example those treated with temozolomide alone.

6 Objectives

6.1 Primary objectives

6.1.1 To evaluate the impact of biopsy versus extensive resection on time to progression after first recurrence of GBM

6.2 Secondary objectives

6.2.1 To evaluate the impact of biopsy versus extensive resection on PFS-6

6.2.2 To assess the impact of biopsy versus extensive resection on quality of life, as measured by KPS

7 Surgery

7.1 Resection

Patients randomized to microsurgical resection will undergo routine, image-guided cytoreduction targeting the contrast-enhancing portion of the lesion on T1-weighted contrast-enhanced MRI. Intraoperative adjuncts, including and not limited to, 5-aminolevulinic acid, intraoperative MRI, and intraoperative mapping techniques, will be included as needed per the primary neurosurgeon. Gliadel®(carmustine) wafers will not be placed.

7.2 Biopsy

Patients randomized to open biopsy will under routine, image-guided open biopsies using a minimal craniotomy and open microsurgical techniques. (This does *not* include stereotactic needle biopsy).

8 Drug treatment: bevacizumab

8.1 Overview

In both treatment arms, patients will be treated post-operatively with bevacizumab. The first treatment will be administered on post-operative day 28.

8.2 Dose

Bevacizumab will be administered at a dose of 10 mg/kg every 2 weeks. Doses will be adjusted if there is a >10% change in weight. While every effort will be made to keep bevacizumab infusions exactly 14 days apart, it is acknowledged that occasionally a dose must be given off schedule due to logistical reasons. A window of ± 4 days for bevacizumab dosing is acceptable.

8.3 Administration

Bevacizumab will be administered intravenously as per current institutional guidelines, with associated pre-medications where required.

8.4 Duration of treatment

Treatment with bevacizumab will continue until progression of disease or significant toxicity occurs. Treatment may be held for >grade 3 toxicities as defined in section 8.5. If the patient does not meet criteria to resume treatment within 28 days of the date of the toxicity, they will be removed from the study treatment.

8.4.1 Description and packaging

Bevacizumab is a humanized IgG1 monoclonal antibody (mAb) that binds all biologically active isoforms of human vascular endothelial growth factor (VEGF) with high affinity. The mAb consists of a human IgG1 framework and the antigen-binding, complementarity-determining regions from the murine anti-VEGF mAb A.4.6.1.16-18. Vials contain bevacizumab with phosphate, trehalose, polysorbate 20, and sterile water for injection.

8.4.2 Administration

Bevacizumab should be administered as a continuous intravenous infusion using a rate regulating device per institutional guidelines. Pre-medications (e.g. ondansetron, diphenhydramine, dexamethasone) will be given as per local guidelines. It should not be administered as an intravenous (IV) push or bolus.

The first dose should be given over a minimum of 90 minutes. If no adverse reactions occur, the second dose should be given over 60 minutes. If no adverse reactions occur, subsequent doses can be given over a minimum of 30 minutes. If infusion-related adverse reactions occur, all subsequent infusions should be administered over the shortest period that was well tolerated.

8.4.3 Storage

Vials contain no preservative and are suitable for single use only.

8.4.4 Supply

Bevacizumab is available commercially.

8.5 Adverse events (AEs)

For studies with participating centers in the USA and registered with the National Cancer Institute (NCI), this is done through the Cancer Therapy Evaluation Program Adverse Event Reporting System (CTEP-AERS). To facilitate reporting, a list of reported and/or potential AEs associated with an agent is provided by the Comprehensive Adverse Event and Potential Risks list (CAEPR). This uses a uniform presentation of events by body system.

In table 8.5 below, these are shown for bevacizumab. In addition to the comprehensive list, a subset subset of AEs is shown which are protocol specific exceptions to expedited reporting to the NCI via CTEP-AERS. These are the Specific Protocol Exceptions to Expedited Reporting (SPEER). This list is based on a recent Radiation Therapy Oncology Group (RTOG) study involving bevacizumab. [14]

AEss listed on the SPEER should be reported expeditiously by investigators to the NCI via CTEP-AERS only if they *exceed* the grade of the event listed in parentheses after the event.

Adverse events reported with bevacizumab			
LIKELY (>20%)	LESS LIKELY (≤ 20%)	RARE (<3%)	SPEER
	Blood and lymphatic system disorders		
	Anemia		3
		Blood and lymphatic system disorders - Other (renal thrombotic microangiopathy)	
	Febrile neutropenia		3
	Cardiac disorders		
	Supraventricular tachycardia		3
		Acute coronary syndrome	
		Ventricular arrhythmia	

Table 8.1: Reported adverse events (continued)

Likely (>20%)	Less likely (≤ 20%)	Rare (<3%)	SPEER
		Ventricular fibrillation	
	Ear and labyrinth disorders		
	Vertigo		
	Gastrointestinal disorders		
	Abdominal pain		3
	Colitis		3
	Constipation		3
	Colitis		3
	Diarrhea		3
	Dyspepsia		2
		Gastrointestinal fistula[1]	
	Gastrointestinal hemorrhage[2]		
	Gastrointestinal obstruction[3]		
		Gastrointestinal perforation[4]	
		Gastrointestinal ulcer[5]	
	Ileus		
	Mucositis oral		3
	Nausea		3
	Vomiting		3
	General disorders and administration site conditions		
	Fatigue		3

[1] Gastrointestinal fistula may include: Anal fistula, Colonic fistula, Duodenal fistula, Esophageal fistula,Gastric fistula, Gastrointestinal fistula, Rectal fistula, and other sites under the GASTROINTESTINAL DISORDERS SOC.

[2] Gastrointestinal hemorrhage may include: Colonic hemorrhage, Duodenal hemorrhage, Esophageal hemorrhage, Esophageal varices hemorrhage, Gastric hemorrhage, Hemorrhoidal hemorrhage, Intraabdominal hemorrhage, Oral hemorrhage, Rectal hemorrhage, and other sites under the GASTROINTESTINAL DISORDERS SOC.

[3] Gastrointestinal obstruction may include: Colonic obstruction, Duodenal obstruction, Esophageal obstruction, Ileal obstruction, Jejunal obstruction, Rectal obstruction, Small intestinal obstruction, and other sites under the GASTROINTESTINAL DISORDERS SOC.

[4] Gastrointestinal perforation may include: Colonic perforation, Duodenal perforation, Esophageal perforation, Gastric perforation, Jejunal perforation, Rectal perforation, Small intestinal perforation, and other sites under the GASTROINTESTINAL DISORDERS SOC

[5] Gastrointestinal ulcer may include: Duodenal ulcer, Esophageal ulcer, Gastric ulcer, and other sites under the GASTROINTESTINAL DISORDERS SOC.

Table 8.1: Reported adverse events (continued)

Likely (>20%)	Less likely (≤ 20%)	Rare (<3%)	SPEER
	Infusion-related reaction		2
	Non-cardiac chest pain		3
	Pain		3
	Immune system disorders		
	Allergic reaction		2
		Anaphylaxis	
	Infections and infestations		
	Infection [6]		3
	Infections and infestations - Other (peri-rectal abscess)		
	Injury, poisoning and procedural complications		
		Gastrointestinal anastomotic leak	
	Wound dehiscence		2
	Investigations		
	ALT increased		3
	AST increased		3
	Blood bilirubin increased		2
	Alkaline phosphatase increased		3
	Cardiac troponin I increased		
	ANC decreased		3
	Weight loss		3
	WCC decreased		3
	Metabolism and nutrition disorders		
	Anorexia		3
	Musculoskeletal and connective tissue disorders		
	Arthralgia		3
	Musculoskeletal and connective tissue disorder - Other (bone metaphyseal dysplasia)[7]		

[6] Infection may include any of the 75 infection sites under the INFECTIONS AND INFESTATIONS SOC.

[7] Metaphyseal dysplasia was observed in young patients who still have active epiphyseal growth plates.

Table 8.1: Reported adverse events (continued)

Likely (>20%)	Less likely (≤ 20%)	Rare (<3%)	SPEER
	Myalgia		3
	Osteonecrosis of jaw [8]		
Nervous system disorders			
	Dizziness		2
	Headache		3
		Intracranial hemorrhage	
		Ischemia cerebrovascular	
	Peripheral sensory neuropathy [9]		
		Reversible posterior leukoencephalopathy syndrome (RPLS)	
	Syncope		
Renal and urinary disorders			
		Acute kidney injury	
	Hematuria		3
	Proteinuria		2
		Renal and urinary disorders - Other (Nephrotic Syndrome)	
		Urinary fistula	
Reproductive system and breast disorders			
Reproductive system and breast disorders - Other (ovarian failure) [10]			

[8] Cases of osteonecrosis of the jaw have been reported in cancer patients in association with bevacizumab treatment, the majority of whom had received prior or concomitant treatment with IV bisphosphonates.

[9] Increased rate of peripheral sensory neuropathy has been observed in trials combining bevacizumab and chemotherapy compared to chemotherapy alone.

[10] Ovarian failure, defined as amenorrhea lasting 3 or more months with follicle-stimulating hormone (FSH) elevation (≥ 30 mIU/mL), was increased in patients receiving adjuvant bevacizumab plus mFOLFOX compared to mFOLFOX alone (34% vs. 2%). After discontinuation of bevacizumab, resumption of menses and an FSH level <30 mIU/mL was demonstrated in 22% (7/32) of these women. Long term effects of bevacizumab exposure on fertility are unknown.

Table 8.1: Reported adverse events (continued)

Likely (>20%)	Less likely (≤ 20%)	Rare (<3%)	SPEER
		Vaginal fistula	
	Vaginal hemorrhage		3
	Respiratory, thoracic and mediastinal disorders		
	Allergic rhinitis		3
		Bronchopleural fistula	
		Bronchopulmonary hemorrhage	
	Cough		3
	Dyspnea		2
	Epistaxis		3
	Hoarseness		3
		Respiratory, thoracic and mediastinal disorders - Other (nasalseptal perforation)	
		Respiratory, thoracic and mediastinal disorders - Other (tracheo-esophageal fistula)	
	Skin and subcutaneous tissue disorders		
	Pruritis		2
	Rash maculo-papular		2
	Urticaria		2
	Vascular disorders		
Hypertension			3
	Thromboembolic event		3
		Vascular disorders - Other (arterial thromboembolic event) [11]	

[11] Arterial thromboembolic event includes visceral arterial ischemia, peripheral arterial ischemia, heart attack, and stroke.

The following have also been reported with bevacizumab but causality has not been established.

Other adverse events reported with bevacizumab	
BODY SYSTEM	ADVERSE EVENT
Blood and lymphatic system disorders	Blood and lymphatic system disorders - Other (idiopathic thrombocytopenia purpura)
	Disseminated intravascular coagulation
Cardiac disorders	Pericardial effusion
General disorders conditions	Gait disturbance
	Sudden death (not otherwise specified)
Hepatobiliary disorders	Hepatic failure
Infections and infestations	Infections and infestations - Other (aseptic meningitis)
Investigations	Platelet count decreased
Metabolism and nutrition disorders	Hyponatremia
Musculoskeletal and connective tissue disorders	Musculoskeletal and connective tissue disorder - Other (aseptic necrotic bone)
	Musculoskeletal and connective tissue disorder - Other (myasthenia gravis)
Nervous system disorders	Dysgeusia
	Peripheral motor neuropathy
	Seizure
Psychiatric disorders	Confusion
Respiratory, thoracic and mediastinal disorders	Adult respiratory distress syndrome
Pneumonitis	
	Pneumothorax
	Pulmonary hypertension
Skin and subcutaneous tissue disorders	Palmar-plantar erythrodysesthesia syndrome
	Skin ulceration

8.5.1 Bevacizumab Dose Modifications

There will be no dose reduction for bevacizumab. Treatment should be interrupted or stopped for certain adverse events, as described in table 8.5.1. If bevacizumab is interrupted for >8 weeks, the patient should stop bevacizumab treatment on protocol.

Treatment Modification for Bevacizumab-Related Adverse Events

Event	Grade	Action to be taken
Allergic reactions or Acute infusional reactions/ cytokine release syndrome	Grade 1-3	If infusion-related or allergic reactions occur, premedications should be given with the next dose and infusion time may not be reduced for the subsequent infusion.<br>For patients with grade 3 reactions, bevacizumab infusion should be stopped and not restarted on the same day. At the physicians discretion, bevacizumab may be permanently stopd or re-instituted with premedications and administered patient should be closely monitored for a duration comparable to or longer than the duration of the previous reactions.
	Grade 4	Stop bevacizumab
Arterial Thrombosis: Cardiac ischemia/ infarction, CNS ischemia (TIA, CVA), any peripheral or visceral arterial ischemia/ thrombosis	Grade 2	If new or worsened since bevacizumab therapy: Stop bevacizumab
	Grade 3-4	Stop bevacizumab
Venous Thrombosis	Grade 3 or asymptomatic grade 4	Hold bevacizumab treatment. If the planned duration of full-dose anticoagulation is <2 weeks, bevacizumab should be held until the full-dose anticoagulation period is over.<br>If the planned duration of full-dose anticoagulation is >2 weeks, bevacizumab may be resumed during the period of full-dose anticoagulation if *all* of the criteria below are met:

Table 8.3: Treatment modifications (continued)

Event	Grade	Action to be taken
		– The subject must have an in-range INR (usually 2-3) on a stable dose of warfarin or be on a stable dose of heparin prior to restarting bevacizumab
		– The subject must not have pathological conditions that carry high risk of bleeding (eg, tumor involving major vessels or other conditions)
		– The subject must not have had hemorrhagic events while on study
		If thromboemboli worsen/recur upon resumption of study therapy, stop bevacizumab
	Grade 4 (symptomatic)	Stop bevacizumab
Hypertension [12]	*Treat with antihypertensive medication as needed. The goal of BP control should be consistent with general medical practice.*	
	Grade 1	Consider increased BP monitoring
	Grade 2 asymptomatic	If diastolic BP <100 mmHg: begin anti-hypertensive therapy and continue bevacizumab
	Grade 2-3	If symptomatic or diastolic BP >100 mmHg: hold bevacizumab until symptoms resolve and BP <160/90mmHg
	Grade 4	Stop bevacizumab
Congestive Heart Failure	Grade 3-4	Stop bevacizumab
Proteinuria	*Proteinuria should be monitored by urine analysis for urine protein creatinine (UPC) ratio prior to every other dose of bevacizumab*	
	UPC ratio <3.5	Continue bevacizumab
	UPC ratio $\geq$ 3.5	Hold bevacizumab until UPC <3.5

[12] Definitions

grade 1 asymptomatic, transient (<24 hours) increase by >20 mmHg (diastolic) or to >150/100 if previously within normal limits (WNL); intervention not indicated

grade 2 recurrent or persistent (>24 hours) or symptomatic increase by >20 mmHg (diastolic) or to >150/100 if previously WNL; monotherapy may be indicated

grade 3 requiring more than one drug or more intensive therapy than previously

grade 4 life threatening (eg, hypertensive crisis)

Table 8.3: Treatment modifications (continued)

Event	Grade	Action to be taken
	Grade 4 or nephrotic syndrome	Stop bevacizumab
Hemorrhage (CNS or pulmonary)	Grade 2-4	Stop bevacizumab
Hemorrhage (non-CNS; non-pulmonary)	Grade 3	Patients receiving full-dose anticoagulation should stop bevacizumab
		For patients not on full-dose anticoagulation, hold bevacizumab until all of the following criteria are met:<br>- the bleeding has resolved and Hb is stable<br>- there is no bleeding diathesis that would increase the risk of therapy<br>- there is no anatomic or pathologic condition that could increase the risk of hemorrhage recurrence.<br>Patients who experience recurrence of grade 3 hemorrhage should stop study therapy
	Grade 4	Stop bevacizumab
RPLS or PRES	*Hold bevacizumab if symptoms/signs suggestive of RPLS; subsequent management should include MRI scans and control of hypertension. Stop bevacizumab upon diagnosis of RPLS*	
Wound dehiscence requiring medical or surgical intervention		Stop bevacizumab
GI perforation, GI leak or fistula		Stop bevacizumab
Bowel obstruction	Grade 2 requiring medical intervention	Hold bevacizumab until complete resolution, with a minimum of 4 weeks after surgery.
	Grade 3-4	Hold bevacizumab until complete resolution<br>If surgery is required, patient may restart bevacizumab after full recovery from surgery, and at investigators discretion
Other unspecified bevacizumab-related AEs (except controlled nausea/ vomiting)	Grade 3	Hold bevacizumab until symptoms resolve to $\leq$ grade 1
	Grade 4	Stop bevacizumab

Table 8.3: Treatment modifications (continued)

Event	Grade	Action to be taken
		Upon consultation with the study chair, resumption of bevacizumab may be considered if a patient is benefiting from therapy and the grade 4 toxicity is transient, has recovered to $\leq$ grade 1 and unlikely to recur with retreatment

9 Monitoring

Patients will follow up with their Oncologist at monthly intervals. MRIs will be repeated every 2 months, or more frequently if there are new symptoms or signs concerning for disease progression. KPS will be assessed at each visit.

10 Treatment at time of progression

At time of subsequent progression, the patient will be re-evaluated at our multidisciplinary conference. Decisions will be individualized but will include the options of further surgery, further RT, a change in chemotherapy, treatment with electrical fields (NovoTTF ®) and Palliative care.

11 Statistical considerations

11.1 Sample size and power justification

The primary objective of this study is to determine whether extensive resection plus bevacizumab (experimental arm) will improve the overall survival compared to open biopsy plus bevacizumab (control arm). The division into experimental and control arms is somewhat arbitrary as both are established treatements at the time of progression and neither has clearly been shown to be superior.

Essentially, this is a Phase III design. The randomization of experimental and control arms is $1:1$. The null hypothesis is that the median PFS for both arms will be 18 weeks, based on data from two Phase II trials of bevacizumab at time of recurrence. [9][12] The alternative hypothesis is that patients treated with *an attempt at complete resection* plus bevacizumab will have an improvement in overall survival to 23 weeks (hazard ratio (HR) of 1.3). This is based on a review of existing literature (retrospective series) on this subject, which is detailed in the article describing this trial.

Following the methods of Therneau, we assume that the survival distributions will be exponential. [15] The HR is thus the reciprocal of the ratio of median survival times. We aim for a power of 80% and a significance level of

0.05 (two-sided). This would result in a sample size of 456 (assuming all patients are treated as assigned and followed until to to progression). A typical range of power and sample sizes is illustrated in table 11.1 for the above significance level, following the method of Schoenfeld. [13]

Table 11.1: Sample size for $p < 0.05$ (two-sided)

	Hazard ratio (HR)		
Power	1.2	1.4	2.0
70%	742	218	51
80%	945	277	65
90%	1264	370	87.5

Given the risks associated with overestimating the effect size in the study, which would lead to the study being underpowered, we prefer the more conservative margin of a HR of 1.2. Thus, we aim for for a sample size of 945.

Following a review of cases over the preceding 3 years (2009-2012), we estimate that our institution sees 50-70 cases/year of first recurrence of GBM who had previously received standard of care (the Stupp regimen, i.e. concurrent RT and TMZ followed by adjuvant TMZ). [4] This numers seen appear to be stable over this time period. We will include those who were receiving this regimen as part of another trial. We estimate that 60-70% will be eligible for entry, and agreeable, to participation in the study, giving approximately 40 entrants/year.

Given the more optimistic threshold of a HR of 1.3, the trial would take 11 years to accrue. With the more conservative HR of 1.2, the trial would need to run for *c.* 23 years.

11.2 Patient accrual

This study is projected to accrue 3-4 patients/month. If the average accrual is <2 patients/month (6 months after trial activation), the study will be reevaluated with respect to feasibility.

11.3 Patient selection and randomization

All cases will be reviewed at our weekly weekly Multidisciplinary Central Nervous System Tumor Conference. Enrollment will be considered for all patients meeting the eligibility criteria. 3 Patients will be stratified at the time of enrollment; this will be followed by radomization, as shown in the study schema 2.

11.4 Analysis plans

In general, comparisons between groups will have a significance (type I error) of $p < 0.05$ (2-sided).

Progression-free and overall survival rates will be estimated using the Kaplan-Meier method. [11] Differences between treatment arms will be tested with the log-rank test. [10] PFS-6 and mOS will be measured from the date of randomization to the date of death or, otherwise, to the last follow-up date on which the patient was known to be alive.

Proportions/ rates will be modelled using an exact binomial distribution, with 95% confidence intervals. These are: PFS-6, objective response, grade 3+ toxicities and acute or delayed CNS toxicities. For secondary endpoints, no adjustament is planned for multiple comparisons as these are exploratory tests.

MOS and PFS-6 will also be modelled with multivariate analyses using the Cox proportional-hazards model. The planned covariates of interest are:

- Protocol treatment (biopsy or extensive resection)
- Age
- Tumor volume at time of recurrence
- KPS
- NIH Recurrent GBM scale (NRG).
  Number of critical areas of cortex involved (0-3)[13]
- Time since original diagnosis

The assumption of roportional hazards will be checked using residuals and time-varying coefficients. If the data clearly do not follow proportional hazards, other statistical models will be used to fit the data instead. Possible alternatives are to use the stratified Cox proportional hazard model, accelerated failure model, or to partition the time axis into sections where proportional hazards assumption holds.

11.5 Interim toxicity and futility analysis

Interim analysis will be undertaken on an annual basis following the methods of O'Brien and Fleming. [16] The study will be terminated of study should these initial results indicate a marked superiority of one treatment over the other.

[13] These are

- Motor
- Speech
- MCA i.e. referring to affected areas adjacent to the M1/M2 areas of the Middle Cerebral Artery

11.6 Significance Testing for Final Analysis

The final analysis will be performed on an intention-to-treat basis, such that all eligible cases on the study will be included in the arm to which they were randomized, regardless of what treatment the patients actually received. The analysis to report the final results of treatment comparison between the experimental arm and the control arm will be undertaken once the required sample size has been accrued. If the p value is less than protocol-specified 0.05 (two-sided), the study Statistician will reject the null hypothesis and conclude that observing such a dataset would be highly unlikely if the outcome in both arms was really similar. Thus the data would support the hypothesis that the experimental arm improves progression-free survival. The final report will include all information reported in the interim analyses as well as treatment compliance and adverse events.

12 Data collection

12.1 Data storage and analysis

The Research Nurse assigned to this study will tabulate, store, and secure data in a single password-protected Excel® database located on a password-protected computer in their locked office. Data analysis will be completed by the Principal Investigator and Statistician.

12.2 Data safety monitoring plan

The Data Safety Monitoring Plan is written to ensure the safety of the participants and verifying the validity and integrity of the data. All adverse events will be reported to the institutional review board (IRB) and FDA in an expedited manner within 10 days of occurrence. The investigator will continue to follow or obtain documentation of the resolution course of such an event.

12.3 Ethical considerations and protection of human subjects

For patients that meet the inclusion criteria, informed consent will be obtained at the time of consent for brain tumor surgery. The option to enter the trial will be presented to the patient, if he/she is able to consent, or to the legal guardian/durable power of attorney in all other circumstances.

For all patients, the risks, benefits and alternatives of the trial will be discussed separately from those associated with the planned operation. Refusal to participate in the trial will not affect management strategies or therapeutic options.

12.4 Potential risks

The risks of study inclusion are similar to the risks of open neurosurgical tumor resection. This includes, but is not limited to, death, coma, infection, blood loss, and permanent neurological deficit.

12.5 Potential benefits

While the study provides no direct benefit to the patient, its findings may clarify the value of greater extent of resection for patients with recurrent glioblastoma.

12.6 What other alternatives are there?

If you decide not to participate in this research study, this will not affect the care to which you are entitled and will not influence your doctor in any way. If you decide not to participate, your doctor will discuss alternative treatment options with you. Some of these options may include open surgery, open biopsy, needle biopsy, and observation.

12.7 Protecting confidentiality

Each consented participant will be assigned a study identification number. All data will be collected and stored under this identification number, with no other identifying information such as name, date of birth, or social security number. All collected data will be kept on premises of the Hospital in a password-protected desktop computer located in a locked office accessible only to the Principal Investigator and Study Co-ordinator. A list of corresponding medical record numbers will be similarly tabulated and protected.

12.8 Costs

There will be no additional supply or equipment costs incurred to the patient or the hospital by this study. All proposed therapies are within the acceptable standards of current care options and will be billed to the patients insurance company, as would otherwise be the case.

APPENDIX

A Sample consent form

Why am I being asked to participate in this research study?

You have been identified as a possible candidate for participation in a clinical research study called 'Biopsy vs Extensive Resection for first recurrence of Glioblastoma: The BERG trial'. A research study is performed when physicians try find new ways to diagnose and/or treat illness. Since this research study is experimental and the anticipated results have not been proven, you need to know enough about the risks and benefits to decide if you want to participate. This process is called informed consent.

The Research Nurse working with the Principal Investigator for this research study, will discuss this study with you in detail. This Informed Consent document explains what will be expected of you and what risks or benefits you may anticipate if you agree to participate. You should read this document very carefully and ask as many questions as you need to fully understand what your involvement in this study means. Please understand that by signing this document you agree to participate in this experimental study.

How many people will take part in this study?

This Hospital is sponsoring this research study. It is being conducted only at this Hospital. A target of a total of 945 patients will participate.

Why is this study being done?

This research study is designed to better understand the value of greater extent of resection when your tumor shows signs of recurrence. By randomizing patients to extensive resection vs. open biopsy, we will determine whether one has more clinical value than the other. This remains an unresolved question. All proposed treatments are already FDA-approved and are not experimental.

What is involved in this study?

If you choose to participate in this study, you will be selected for either open biopsy (where a small hole is made in the skull to take a piece of the abnormal tissue and make a diagnosis) or microsurgical resection (where an opening in the skull is made to expose all the abnormal tissue and remove everything that is safe). These are both currently standard therapy options for patients with your diagnosis. The purpose of the study is to determine which provides more clinical benefit. Following surgery, you will recover as you would normally and then, if the tissue that is removed is diagnosed as recurrent tumor, receive IV bevacizumab, an anti-tumor agent that commonly given to patients at the time of recurrence. Your clinical follow-up after this will be no different than it

would be otherwise, with routine appointments to see your Neuro-Oncologist and Neurosurgeon at regular intervals.

How long will I be on the study?

If you agree to take part in this study, we will continue up with you for at least 3 years. As stated above, no additional appointments will be needed during this time.

What are the risks of this study?

The risks of this study are similar to those that you would be faced with during routine management of your tumor.

Risks associated with brain surgery, include death, neurological problems such as weakness, difficulty seeing or using language, blood loss, and infection.

Risks associated with bevacizumab include wound infection, hematological abnormalities (low blood counts), and gastrointestinal problems.

Are there benefits to taking part in this study?

This study involves treatments which would be available to you outside the context of the study. However there may be benefits either to having a surgical resection or to having a biopsy alone in terms of quality of life and survival. Also, the knowledge gained from this study may benefit others in the future.

What other options are there?

If you decide not to participate in this research study, this will not affect the care to which you are entitled and will not influence your doctor in any way. If you decide not to participate, your doctor will discuss alternative treatment options with you. Some of these options may include open surgery, open biopsy, needle biopsy, and observation.

How will I be informed of additional information or new findings?

If you decide to participate, your doctor will keep you informed of any new findings that are discovered during the course of this study which may affect your continued willingness to participate in this study.

What about confidentiality?

Every attempt will be made to keep your participation in this research study and your records confidential. However, representatives from the Department of Health and Human Services (DHHS), from the FDA and the Hospital may

inspect your research records to evaluate the results of this study. The results of this research study may also be published in scientific journals and/or may be presented at scientific meetings, but your identity will not be revealed.

A description of this clinical trial will be made available on ClinicalTrials.gov, as is required by U.S. Law. This Web site will not include information that can identify you. At most, the Web site will include a summary of the results. You can search this Web site at any time.

What are the costs?

There are no additional costs for you that are associated with this trial. As always, you understand that you will be responsible for payment of any bills that your insurance may refuse to pay due to your participation in this research study.

What happens if I am injured while participating in the study?

If you experience an injury or adverse event, please contact the Principal Investigator at 123 456 78910. If the investigator determines that the injury or adverse event is due to your participation in this research, this will be treated, although you are always responsible for the financial costs associated with your care. This does not waive your rights in the event of negligence.

What are my rights as a participant?

You understand that your participation in this research study is voluntary and that you are free to withdraw from this study at any time. If you decide not to participate, your doctor will not hold it against you in any way. If you decide to withdraw from this study at a later date, please notify the Principal Investigator of your decision and to discuss your alternative treatment options.

You understand that your doctor may also stop your participation on this study if it is felt to be in the best interest of your health. This may be done without your consent, but alternative treatment options will be discussed with you.

Whom do I call if I have questions?

If you have any questions about this research study or your participation in it, please feel free to ask them now. If you think of questions later or have concerns about your participation, please call the Principal Investigator at 123 456 78910.

If you have any questions about your rights as a research subject or about the Institutional Review Board for Human Research (IRB) at the Hospital. The board has reviewed this Informed Consent document for compliance with federal guidelines. They may be contacted at: Institutional Review Board

Hospital
Address
Phone no. You are voluntarily deciding whether or not to participate in the

research study described in this consent form. Your signature below indicates that you have read and understand the information provided, and that you have decided to participate. You will receive a copy of the signed informed consent document.

Patient/ representative signature

____
Printed name

____
Signature

____
Date

____
Signature of Legally Authorized Representative

____
Relationship to patient

____
Reason patient is unable to sign

____
Date

____
Signature of Interpreter (If applicable)

Principal investigator's signature

Printed name

Signature

Date

B Rights of humans subjects in medical experiments

Any person who is requested to consent to participate as a subject in a research study involving a medical experiment or who is requested to consent on behalf of another has the right to:

1. Be informed of the nature and purpose of the experiment.
2. Be given an explanation of the procedures to be followed in the medical experiment, and any drug or device to be utilized
3. Be given a description of any attendant discomforts and risks reasonably to be expected from the experiment
4. Be given an explanation of any benefits to the subject reasonable to be expected from the experiment, if applicable
5. Be given a disclosure of any appropriate alternative procedures, drugs or devices that might be advantageous to the subject, and their relative risks and benefits
6. Be informed of the avenues of medical treatment, if any available to the subject after the experiment if complications should arise
7. Be given an opportunity to ask any questions concerning the experiment or the procedure involved
8. Be instructed that consent to participate in the medical experiment may be withdrawn at any time and the subject may discontinue participation in the medical experiment without prejudice
9. Be given a copy of any signed and dated written consent form used in relation to the experiment
10. Be given the opportunity to decide to consent or not to consent to a medical experiment without the intervention of any element of force, fraud, deceit, duress, coercion or undue influence on the subjects decision

I attest that I, or my representative, discussed this study with the above named participant or legal representative. This person had enough time to consider this information, had an opportunity to ask questions, and voluntarily agreed to participate in this study.

C Karnofsky performance scale

100 Normal; no complaints; no evidence of disease

90 Able to carry on normal activity; minor signs or symptoms of disease

80 Normal activity with effort; some sign or symptoms of disease

70 Cares for self; unable to carry on normal activity or do active work

60 Requires occasional assistance, but is able to care for most personal needs

50 Requires considerable assistance and frequent medical care

40 Disabled; requires special care and assistance

30 Severely disabled; hospitalization is indicated, although death not imminent

20 Very sick; hospitalization necessary; active support treatment is necessary

10 Moribund; fatal processes progressing rapidly

0 Dead

References

[1] Louis DN, Ohgaki H, Wiestler OD, et al. The 2007 WHO classification of tumours of the central nervous system. Acta neuropathologica 2007, 114:97-109. 11

[2] CBTRUS Statistical Report: Primary Brain and Central Nervous System Tumors Diagnosed in the United States in 2004-2008, 2012. 11

[3] Taal W, Brandsma D, de Bruin HG, et al. Incidence of early pseudo-progression in a cohort of malignant glioma patients treated with chemoirradiation with temozolomide. Cancer 2008, 113:405-410. 11

[4] Stupp R, Mason WP, van den Bent MJ, et al. Radiotherapy plus concomitant and adjuvant temozolomide for glioblastoma. NEJM 2005, 352:987-996. 11, 24

[5] Wong ET, Hess KR, Gleason MJ, et al. Outcomes and prognostic factors in recurrent glioma patients enrolled onto Phase II clinical trials. J Clin Oncol 1999, 17:2572-2578. 11

[6] Easaw JC, Mason WP, Perry J, et al. Canadian Glioblastoma Recommendations Committee. Canadian recommendations for the treatment of recurrent or progressive glioblastoma multiforme. Curr Oncol 2011, 18(3):126-136. 11

[7] NCCN Guidelines Version 2.2013. Anaplastic gliomas/ glioblastoma. Available online 11

[8] Brandes AA, Bartolotti M, Franceschi E. Second surgery for recurrent glioblastoma: advantages and pitfalls. Expert Rev Anticancer Ther 2013 13(5):583-587. 11

[9] Friedman HS, Prados MD, Wen PY et al. Bevacizumab alone and in combination with irinotecan in recurrent glioblastoma. J Clin Oncol, 2009 27(28):4733-4740. 12, 23

[10] Mantel N. Evaluation of survival data and two new rank order statistics arising in its consideration. Cancer Chem Rep 1966 5:163-170. 25

[11] Kaplan EL, Meier P. Nonparametric estimation from incomplete observations. J Am Stat Assoc 1958 53:457-481 25

[12] Kreisl TN, Kim L, Moore K et al. Phase II trial of single-agent bevacizumab followed by bevacizumab plus irinotecan at tumor progression in recurrent glioblastoma. J Clin Oncol, 2009 27(5):740-745. 12, 23

[13] Schoenfeld D. Sample-size formula for the proportional-hazards regression model. Biometrics 1983, 39:499-503. 24

[14] Gilbert M. Phase III Double-Blind Placebo-Controlled Trial of Conventional Concurrent Chemoradiation and Adjuvant Temozolomide Plus Bevacizumab versus Conventional Concurrent Chemoradiation and Adjuvant Temozolomide in Patients with Newly Diagnosed Glioblastoma. Available online 14

[15] Therneau T, Grambsch P. Modeling Survival Data: Extending the Cox Model. Springer: New York; 2000. 23

[16] Obrien P, Fleming T. A Multiple Testing Procedure for Clinical Trials. Biometrics 1979, 35:549-556. 25
