# Supplementary material for: Biopsy vs. extensive resection for first recurrence of glioblastoma: is a prospective clinical trial warranted?
Source: BMC Res Notes. 2015 Sep 4;8:414. doi: 10.1186/s13104-015-1386-3 (PMC4560929; doi:10.1186/s13104-015-1386-3)
Supplement: Supplementary file 2 — Additional file 2. LaTeX code used to generate the BERG trial protocol. This is provided so that others can modify the protocol or use elements from it. [file 13104_2015_1386_MOESM2_ESM.pdf]

```latex
\documentclass{article}
%
% for meaningless text
\usepackage{lipsum}
% for named colors
\usepackage[table]{xcolor}
% for \cellcolor
\usepackage{colortbl}
% for tables > 1 page
\usepackage{longtable}
\usepackage{hhline}
% for matrix (used in tikz plot)
\usepackage{array}
% multirow in tables
\usepackage{multirow}
% for 2-column format
\usepackage{multicol}
% change table numbering
\usepackage{chngcntr}
\counterwithin{table}{section}
% increase space between rows
\renewcommand{\arraystretch}{1.3}
\usepackage{booktabs}
%
% for 2-column entries in table
\newcommand{\myTwoCol}[1]{
  \textsl{#1}}
%
% for plotting
\usepackage{tikz}
\usetikzlibrary{arrows, shapes}

% version history
\usepackage{vhistory}
\newcommand{\docTitle}{Biopsy vs Extensive Resection for first
  recurrence of Glioblastoma: The \textbf{BERG} trail}
% for author - used by version history
\newcommand{\CD}{Christopher Dardis}
%
% place hyperref close to end
\usepackage[pagebackref]{hyperref}
\hypersetup{
  pdftitle = {\docTitle},
  pdfkeywords = {\docTitle,
    Version \vhCurrentVersion from \vhCurrentDate},
  pdfauthor = {\vhAllAuthorsSet},
  filebordercolor=cyan,
  linkbordercolor=cyan,
  urlbordercolor=cyan,
  runbordercolor=cyan
}
\usepackage{footnotebackref}
%% make footnotebackref work with longtable
\usepackage{etoolbox}
\patchcmd{\footnote}{\edef}{\xdef}{}{\errmessage{failed to patch}}
\makeatletter
\renewcommand\@makefntext[1]{%
    \edef\@makefnmark{%
        \noexpand\mbox{\noexpand\textsuperscript{\noexpand\normalfont%
        \noexpand\hyperref[\BackrefFootnoteTag]{\noexpand\@thefnmark}}}\noexpand\,}%
    \BHFN@OldMakefntext{#1}}%
\makeatother
%
% acronym needs go after hyperref
\usepackage[acronym, nomain, toc=true, numberline=true]{glossaries}
\makeglossaries
%
\begin{document}

\title{\docTitle}
\author{\vhListAllAuthorsLongWithAbbrev}
\date{Version \vhCurrentVersion\ from \vhCurrentDate}
\maketitle

\begin{versionhistory}
\vhEntry{0.1}{10/18/2013}{CD}{Created}
\vhEntry{0.2}{7/01/2014}{CD}{Updated}
\end{versionhistory}

%\title{Biopsy vs. Extensive Resection for first recurrence of Glioblastoma: The BERG trail}
```

```latex
%\maketitle

\newpage

% show only sections in contents
\setcounter{tocdepth}{1}
\tableofcontents
%
% autoformat numbers to 5 levels depth
% (avoids numbering subsubsections)
\setcounter{secnumdepth}{6}

\newpage

\section{Study team}

\begin{multicols}{2}

\subsection{Principal Investigator (Neurosurgery)}
\lipsum[66]

\subsection{Co-Investigator (Neuro-Oncology)}
\lipsum[66]

\columnbreak

\subsection{Neurosurgery:}
\lipsum[75]

\subsection{Neuro-Oncology:}
\lipsum[75]

\subsection{Radiation Oncology:}
\lipsum[75]

\end{multicols}

\newpage

\section{Schema}
\label{sec:sc}

\bigskip

% Define block styles
\tikzstyle{blockC} = [rectangle, draw, fill=blue!5, align=center,
text centered, rounded corners, minimum height=6em]
\tikzstyle{blockL} = [rectangle, draw, fill=blue!5, align=left,
text centered, rounded corners, minimum height=6em]
\tikzstyle{line} = [draw, -latex']

\begin{tikzpicture}
  \matrix [column sep=5mm, row sep=7mm]{
    % row 1
    &
    \node [blockC] (e1) { \textbf{Enroll:}\\ \\
      First recurrence of GBM \\ Age $\geq$ 18  \\ KPS
      $\geq$ 70  \\ \textgreater 1 month  since RT\\
      Prior conventional treatment\\ Decompression not required \\
      Resection possible
    }; & \\
    % row 2
    & \node [blockL] (s1) {\quad \quad \quad \quad \quad \quad \quad
      \textbf{Stratify: NRG scale} \\ \\
      \quad \quad \quad \quad \quad \quad \quad
      \textbf{0 vs. 1 vs. 2 vs. 3}\\ \\
      \textbullet \ KPS $\leq$ 80\\
      \textbullet \ tumor volume $\geq 50 cm^{-3}$\\
      \textbullet \ $\geq$ 2 cortical areas involved:\\
      \quad $\circ$ Motor \\
      \quad $\circ$ Speech \\
      \quad $\circ$ MCA (affected areas adjacent to \\
      \quad \quad the M1/M2 areas of the Middle Cerebral Artery)
    }; & \\
    % row 3
    & \node [blockC] (r1) {\textbf{Randomize}
    }; & \\
    % row 3
    & \node[inner sep = 0pt] (dummy) {}; & \\
    % row 5
    \node [blockC] (b1) {Open biopsy \\ \\ Bevacizumab}; &
```

```latex
    &\node [blockC] (b2) {Resection \\ \\ Bevacizumab}; \\
  };
  %
  \draw[->] (e1) -- (s1);
  \draw[->] (s1) -- (r1);
  \draw[-] (r1) -- (dummy);
  \draw[->] (dummy) -| (b1);
  \draw[->] (dummy) -| (b2);

\end{tikzpicture}

\newpage

\newacronym{gbm}{GBM}{glioblastoma multiforme}
\newacronym{mri}{MRI}{magnetic resonance imaging}
\newacronym{ct}{CT}{computer automated tomography}
\newacronym{kps}{KPS}{Karnofsky performance status}
\newacronym{wcc}{WCC}{white cell count}
\newacronym{anc}{ANC}{absolute neutrophil count}
\newacronym{hb}{Hb}{hemoglobin}
\newacronym{ast}{AST}{aspartate transaminase}
\newacronym{alt}{ALT}{alanine aminotransferase}
\newacronym{uln}{ULN}{upper limit of normal (defined for institution)}
\newacronym{inr}{INR}{international normalized ratio}
\newacronym{egfr}{eGFR}{estimated glomerular filtration rate (MDRD method)}
\newacronym{bhcg}{bHCG}{beta human chorionic gonadotropin}
\newacronym{ivig}{ivIg}{intravenous immune globulin}
\newacronym{vegf}{VEGF}{vascular endothelial growth factor}
\newacronym{bsa}{BSA}{body surface area}
\newacronym{rt}{RT}{radiation therapy}
\newacronym{bp}{BP}{blood pressure}
\newacronym{rpls}{RPLS}{reversible posterior leukoencephalopathy syndrome}
\newacronym{pres}{PRES}{posterior reversible leukoencephalopathy syndrome}
\newacronym{gi}{GI}{gastrointestinal}
\newacronym{nos}{NOS}{not otherwise specified}
\newacronym{nci}{NCI}{National Cancer Institute}
\newacronym{tmz}{TMZ}{temozolomide}
\newacronym{mos}{mOS}{median overall survival}
\newacronym{pfs6}{PFS-6}{progression free survival at 6 months}
\newacronym{ctepaers}{CTEP-AERS}{Cancer Therapy Evaluation Program Adverse Event Reporting System}
\newacronym{irb}{IRB}{institutional review board}
\newacronym{ae}{AEs}{adverse events}
\newacronym{mab}{mAb}{monoclonal antibody}
\newacronym{rtog}{RTOG}{Radiation Therapy Oncology Group}
\newacronym{speer}{SPEER}{Specific Protocol Exceptions to Expedited
  Reporting}
\newacronym{caepr}{CAEPR}{Comprehensive Adverse Event and Potential
  Risks list}
\newacronym{iv}{IV}{intravenous}
\newacronym{dhhs}{DHHS}{Department of Health and Human Services}
\newacronym{fda}{FDA}{Food and Drug Administration}
\newacronym{fsh}{FSH}{follicle-stimulating hormone}
\newacronym{cns}{CNS}{central nervous system}
\newacronym{upc}{UPC}{urine protein creatinine}
\newacronym{wnl}{WNL}{within normal limits}
\newacronym{hr}{HR}{hazard ratio}
\newacronym{nrg}{NRG}{NIH Recurrent GBM scale}

\printglossary[type=\acronymtype, title=Abbreviations, toctitle=Abbreviations]

\newpage

\section{Eligibility}
\label{sec:ec}
\begin{Form}
  % optional make all boxes checked as true by default
  % \makeatletter \Fld@checkedtrue \makeatother
  \begin{center}
    \textbf{Eligibility checklist}
  \end{center}

  \begin{enumerate}
  \item \CheckBox-{} History of \gls{gbm}
  \item \CheckBox-{} First recurrence
  \item \CheckBox-{} Age \textgreater 18
  \item \CheckBox-{} \gls{kps} $\geq$ 70
  \item \CheckBox-{} \textgreater 1 month since \gls{rt}
  \item \CheckBox-{} Prior conventional treatment (Stupp regimen)
  \item \CheckBox-{} \textbf{No} need for surgical decompression
  \item \CheckBox-{} Resection possible
  \item \CheckBox-{} \textbf{No} sign diffuse spread of disease or leptomeningeal disease
```

```
  \item 14 days prior to enrollment:

    \begin{enumerate}
    \item \CheckBox-{} History \& physical exam
    \item \CheckBox-{} Brain imaging shows probable signs of progression
      of disease. \gls{mri} scans are preferred but \gls{ct} is
      acceptable if there is a contraindication to the use of \gls{mri}
    \item \CheckBox-{} Steroid dose stable or decreasing for at least 7
      days
    \item \CheckBox-{} \Gls{bp} - systolic \textless 160, diastolic \textless
      90. Use of antihypertensives permitted
    \item \CheckBox-{} \Gls{wcc} \textgreater 3,000/mm \textsuperscript{3}
    \item \CheckBox-{} \Gls{anc} \textgreater 1,500/mm \textsuperscript{3}
    \item \CheckBox-{} Platelets \textgreater 100,000/mm \textsuperscript{3}
    \item \CheckBox-{} \Gls{hb} \textgreater 10 g/dLt
    \item \CheckBox-{} \Gls{ast}, \gls{alt}, bilirubin \textless 3 \gls{uln}
    \item \CheckBox-{} \Gls{inr} \textless 1.5 \gls{uln}
    \item \CheckBox-{} \Gls{egfr} \textgreater 60mL/min/1.73m \textsuperscript{2}
    \item \CheckBox-{} If potentially pregnant - serum \gls{bhcg} negative
    \end{enumerate}

  \item \CheckBox-{} \underline{1 month} prior: \textbf{no} major surgery
  \item \CheckBox-{} \underline{3 months} prior: \textbf{no} history of non-healing wounds,
    ulcers or bone fractures
  \item \underline{6 months} prior:

    \begin{enumerate}
    \item \CheckBox-{} \textbf{No} unstable angina or myocardial infarct
    \item \CheckBox-{} \textbf{No} heart failure requiring hospitalization
    \item \CheckBox-{} \textbf{No} stroke or transient ischemic attack
    \end{enumerate}

  \item \CheckBox-{} \underline{3 years} prior: \textbf{No} other malignancy (not including
    completely excised basal or squamous cell skin cancer)
  \item \CheckBox-{} If sexually active, agrees to practice adequate
    contraception during study and for 6 months thereafter
  \item \CheckBox-{} \textbf{No} major autoimmune condition requiring immune
    suppression e.g. etanercept, infliximab, ivIg
  \item \CheckBox-{} \textbf{No} prior treatment with anti-VEGF targeted
    treatments e.g. bevacizumab, cediranib, vandetanib, aflibercept,
    sunitinib, sorafenib
  \end{enumerate}
\end{Form}

The Eligibility Checklist must be completed in its entirety prior to
registration. The completed, signed, and dated checklist used at
study entry will be retained in the patients study file.

\newpage

\section{Registration}

\subsection{Procedure}
Patient registration will be conducted in accordance with routine
hospital protocol. Enrolled patients will be further registered in a
de-identified database maintained by the Research Nurse.

\subsection{Data collection at registration}

\begin{Form}
  % optional make all boxes checked as true by default
  % \makeatletter \Fld@checkedtrue \makeatother
  \begin{center}
    \textbf{Checklist for study enrollment}
  \end{center}

  \begin{enumerate}
  \item \CheckBox-{} Name of person randomizing case
  \item \CheckBox-{} Eligibility checklist completed
  \item \CheckBox-{} Investigator considers patient eligible
  \item Informed consent:

    \begin{enumerate}
    \item \CheckBox-{} For trial participation
    \item \CheckBox-{} For tissue and blood to be kept for use in
      research to learn about, prevent or treat cancer
    \item \CheckBox-{} To allow someone from this institution to contact
      him or her in the future to take part in more research
    \end{enumerate}

  \item Patient:
```

```latex
    \begin{enumerate}
    \item \CheckBox-{} Initials
    \item \CheckBox-{} Identification number
    \item \CheckBox-{} Gender
    \item \CheckBox-{} Date of birth
    \item \CheckBox-{} \Gls{kps}
    \item \CheckBox-{} Weight
    \item \CheckBox-{} \Gls{bsa}
    \end{enumerate}
  \item Physicians:
    \begin{enumerate}
    \item \CheckBox-{} Neurosurgeon
    \item \CheckBox-{} Oncologist (Neuro-Oncology or Medical Oncology)
    \item \CheckBox-{} Radiation Oncologist
    \end{enumerate}
  \item Dates:

    \begin{enumerate}
    \item \CheckBox-{} First resection
    \item \CheckBox-{} Started radiation
    \item \CheckBox-{} Completed radiation
    \item \CheckBox-{} Started temozolomide
    \item \CheckBox-{} Completed temozolomide
    \item \CheckBox-{} Registered for this trial
    \item \CheckBox-{} Randomized for this trial
    \end{enumerate}
  \end{enumerate}
\end{Form}

\bigskip \bigskip \bigskip
\noindent
\underline{\hspace{0.75\textwidth}}\\
Printed name

\bigskip \bigskip \bigskip
\noindent
\underline{\hspace{0.75\textwidth}}\\
Completed by

\bigskip \bigskip \bigskip
\noindent
\underline{\hspace{0.3\textwidth}}\\
Date

\newpage

\section{Rationale}

\subsection{Background}

\Gls{gbm} (World Health Organization Grade IV
astrocytoma), is the most common primary brain tumor in adults and
portends a very poor prognosis. \cite{Louis07} Its incidence is
estimated to be 3.2/100,000 in the United States. \cite{CBTRUS2012}

Standard therapy for patients with newly diagnosed \gls{gbm} usually
involves maximal surgical resection, followed by \gls{rt} (typically 60 Gray,
given in 30 fractions) with concomitant and adjuvant \gls{tmz}
for at least 6 months. The addition of \gls{tmz} to \gls{rt} has increased
\gls{mos} from 12.1 months to 14.6 months, and
2-year survival from 10\% to 26\%. \cite{Stupp2005}

Recurrence of \gls{gbm} is almost inevitable. A meta-analysis of 8 Phase II
studies involving 225 patients with recurrent \gls{gbm}, \gls{mos}
after disease recurrence was 25 weeks and the \gls{pfs6} was 15\%. \cite{Wong1999}

Accurately diagnosing recurrence remains a
challenge. Pseudo-progression occurs on follow-up imaging in 20-30\%
of patients imaged at 2 months when treated with standard adjuvant
\gls{rt} and \gls{tmz}. \cite{Taal2008}. In order to differentiate tumor
recurrence from radiographic pseudo-progression, a surgical specimen
remains the gold standard.

\subsection{Surgery}

In some cases surgery is essential in order to relieve mass effect
caused by tumor growth. However in most cases, once recurrence of
tumor has been confirmed (typically by frozen-section performed at the
time of surgery), surgery proceeds with the goal of removal of as much
of the remaining tumor as possible.

There has been considerable debate about the merits of such a
```

```latex
strategy. This is reflected, for example, in the Canadian
recommendations for the treatment of recurrent or progressive GBM,
which states
\begin{quote}
  In the absence of level 1 evidence, the decision to re-operate
  should be made according to individual circumstances, in
  consultation with the multidisciplinary team and the patient.
\end{quote} \cite{Easaw2011}

By contrast the National Comprehensive Cancer Network guideline for
recurrent (local) \gls{gbm} favors resection when possible. \cite{NCCN2013}

A recent review of literature on the subject, the most comprehensive
to date (evaluating 11 studies), concluded that there is no
established role for surgery in this setting. \cite{Brandes2013} Age
and \gls{kps} were identified as generally important prognostic predictors
and to a lesser extent, size of tumor.

\subsection{Bevacizumab}

Regardless of the decision on whether to proceed with surgery (of
whatever extent), some form of additional chemotherapy at recurrence
is generally agreed to be worthwhile where possible. Although no one
agent has yet been endorsed by existing guidelines, all of them give
prime consideration of bevacizumab.

Although no phase III trial has been performed to validate this
strategy, it has been approved by the \gls{fda} on the basis of two phase II
studies, both published in 2009. In that by Friedman et al., those
using bevacizumab alone had a \gls{pfs6}
of 43\% and \gls{mos} of 40 (95\% CI 35.6-46.5) weeks. \cite{Friedman2009}
That by Kresyl et al. showed a \gls{mos} of 31 (95\%CI 21-54)
weeks. \gls{pfs6} was
57\% (95\%CI 44-75). \cite{Kresyl2009} This was a favorable outcome
with respect to historical controls, for example those treated with
temozolomide alone.

\section{Objectives}

\subsection{Primary objectives}
\subsubsection{To evaluate the impact of biopsy versus extensive
  resection on time to progression  after first recurrence of \gls{gbm}}

\subsection{Secondary objectives}
\subsubsection{To evaluate the impact of biopsy versus extensive
  resection on \gls{pfs6}}
\subsubsection{To assess the impact of biopsy versus extensive
  resection on quality of life, as measured by \gls{kps}}

\section{Surgery}

\subsection{Resection}
Patients randomized to microsurgical resection will undergo routine,
image-guided cytoreduction targeting the contrast-enhancing portion of
the lesion on T1-weighted contrast-enhanced \gls{mri}.  Intraoperative
adjuncts, including and not limited to, 5-aminolevulinic acid,
intraoperative \gls{mri}, and intraoperative mapping techniques, will
be included as needed per the primary neurosurgeon.
Gliadel\textregistered (carmustine) wafers will not be placed.

\subsection{Biopsy}
Patients randomized to open biopsy will under routine, image-guided
open biopsies using a minimal craniotomy and open microsurgical
techniques. (This does \emph{not} include stereotactic needle biopsy).

\section{Drug treatment: bevacizumab}

\subsection{Overview}
In both treatment arms, patients will be treated post-operatively with
bevacizumab. The first treatment will be administered on post-operative day 28.

\subsection{Dose}
Bevacizumab will be administered at a dose of 10 mg/kg every 2
weeks. Doses will be adjusted if there is a \textgreater 10\% change
in weight. While every effort will be made to keep bevacizumab
infusions exactly 14 days apart, it is acknowledged that occasionally
a dose must be given off schedule due to logistical reasons.
A window of $\pm$ 4 days for bevacizumab dosing is acceptable.

\subsection{Administration}
Bevacizumab will be administered intravenously as per current
institutional guidelines, with associated pre-medications where
required.
```

```latex
\subsection{Duration of treatment}
Treatment with bevacizumab will continue until progression of disease
or significant toxicity occurs. Treatment may be held for \textgreater
grade 3 toxicities as defined in section~\ref{subsec:ae}. If the
patient does not meet criteria to resume treatment within 28 days of
the date of the toxicity, they will be removed from the study
treatment.

\subsubsection{Description and packaging}
Bevacizumab is a humanized IgG1 \gls{mab} that binds all biologically
active isoforms of human \gls{vegf} with high affinity. The \gls{mab}
consists of a human IgG1 framework and the antigen-binding,
complementarity-determining regions from the murine anti-\gls{vegf}
\gls{mab} A.4.6.1.16-18.
Vials contain bevacizumab with phosphate,
trehalose, polysorbate 20, and sterile water for injection.

\subsubsection{Administration}
Bevacizumab should be administered as a continuous intravenous
infusion using a rate regulating device per institutional guidelines.
Pre-medications (e.g. ondansetron, diphenhydramine, dexamethasone) will be given as per local guidelines. It should not
be administered as an \gls{iv} push or bolus.

The first dose should be given over a minimum of 90 minutes. If no
adverse reactions occur, the second dose should be given over 60
minutes. If no adverse reactions occur, subsequent doses can be given
over a minimum of 30 minutes. If
infusion-related adverse reactions occur, all subsequent infusions
should be administered over the shortest period that was well
tolerated.

\subsubsection{Storage}
Vials contain no preservative and are suitable for single use only.

\subsubsection{Supply}
Bevacizumab is available commercially.

\subsection{\Gls{ae}}
\label{subsec:ae}
For studies with participating centers in the USA and registered with
the \gls{nci}, this is done through the \gls{ctepaers}. To facilitate reporting, a list of reported and/or
potential \gls{ae} associated with an agent is provided by the
\gls{caepr}. This uses a uniform presentation of events by body
system.

In table \ref{tab:ae}
below, these are shown for bevacizumab. In addition to the
comprehensive list, a subset subset of \gls{ae} is shown which are
protocol specific exceptions to expedited reporting to the \gls{nci}
via \gls{ctepaers}.  These are the \gls{speer}. This list is based on
a recent \gls{rtog} study involving bevacizumab. \cite{rtog0825}

\Gls{ae}s listed on the
\gls{speer} should be reported expeditiously by investigators to the
\gls{nci} via \gls{ctepaers} only if they \emph{exceed} the grade of the
event listed in parentheses after the event.

\begin{center}
    \label{tab:ae}
  \begin{longtable}{ @{}
      >{\raggedright}p{3cm}
      >{\raggedright}p{5cm}
      >{\raggedright}p{3.5cm}
      c
      @{} }
    \toprule
    \multicolumn{4}{c}{Adverse events reported with
      bevacizumab}\tabularnewline %
    \midrule %
    \textsc{Likely (\textgreater 20\%)} & \textsc{Less likely ($\leq$
      20\%)} & \textsc{Rare (\textless 3\%)} &
    \textsc{SPEER}\tabularnewline %
    \midrule
    \endfirsthead
    \caption{Reported adverse events (continued)} \tabularnewline%
    \midrule %
    \textsc{Likely (\textgreater 20\%)} & \textsc{Less likely ($\leq$
      20\%)} & \textsc{Rare (\textless 3\%)} &
    \textsc{SPEER}\tabularnewline %
    \hhline{----}
    \endhead
```

```
\endfoot
\bottomrule
\endlastfoot
% start of table cells:
\multicolumn{4}{c}{Blood and lymphatic system
  disorders\cellcolor[gray]{0.9}}\tabularnewline %
& Anemia & & 3 \tabularnewline %
& & Blood and lymphatic system disorders - Other (renal thrombotic
microangiopathy) \tabularnewline %
& Febrile neutropenia & & 3\tabularnewline %
\multicolumn{4}{c}{Cardiac disorders
  \cellcolor[gray]{0.9}}\tabularnewline %
& Supraventricular tachycardia & & 3\tabularnewline %
& & Acute coronary syndrome \tabularnewline %
& & Ventricular arrhythmia \tabularnewline %
& & Ventricular fibrillation \tabularnewline
\multicolumn{4}{c}{Ear and labyrinth disorders
  \cellcolor[gray]{0.9}}\tabularnewline %
& Vertigo \tabularnewline %
\multicolumn{4}{c}{Gastrointestinal disorders
  \cellcolor[gray]{0.9}} \tabularnewline %
& Abdominal pain & & 3\tabularnewline %
& Colitis & & 3\tabularnewline %
& Constipation & & 3\tabularnewline %
& Colitis & & 3\tabularnewline %
& Diarrhea & & 3\tabularnewline %
& Dyspepsia & & 2\tabularnewline %
& & Gastrointestinal fistula\footnote{Gastrointestinal fistula may
  include: Anal fistula, Colonic fistula, Duodenal fistula,
  Esophageal fistula,Gastric fistula, Gastrointestinal fistula,
  Rectal fistula, and other sites under the GASTROINTESTINAL
  DISORDERS SOC.} \tabularnewline %
& Gastrointestinal hemorrhage\footnote{Gastrointestinal hemorrhage
  may include: Colonic hemorrhage, Duodenal hemorrhage, Esophageal
  hemorrhage, Esophageal varices hemorrhage, Gastric hemorrhage,
  Hemorrhoidal hemorrhage, Intraabdominal hemorrhage, Oral
  hemorrhage, Rectal hemorrhage, and other sites under the
  GASTROINTESTINAL DISORDERS SOC.} \tabularnewline %
& Gastrointestinal obstruction\footnote{Gastrointestinal
  obstruction may include: Colonic obstruction, Duodenal
  obstruction, Esophageal obstruction, Ileal obstruction, Jejunal
  obstruction, Rectal obstruction, Small intestinal obstruction,
  and other sites under the GASTROINTESTINAL DISORDERS SOC.} &
\tabularnewline %
& & Gastrointestinal perforation\footnote{Gastrointestinal
  perforation may include: Colonic perforation, Duodenal
  perforation, Esophageal perforation, Gastric perforation,
  Jejunal perforation, Rectal perforation, Small intestinal
  perforation, and other sites under the GASTROINTESTINAL
  DISORDERS SOC} & \tabularnewline %
& & Gastrointestinal ulcer\footnote{Gastrointestinal ulcer may
  include: Duodenal ulcer, Esophageal ulcer, Gastric ulcer, and
  other sites under the GASTROINTESTINAL DISORDERS
  SOC.}\tabularnewline %
& Ileus \tabularnewline %
& Mucositis oral & & 3\tabularnewline %
& Nausea & & 3\tabularnewline %
& Vomiting & & 3\tabularnewline %
\multicolumn{4}{c}{General disorders and administration site
  conditions \cellcolor[gray]{0.9}}\tabularnewline %
& Fatigue & & 3\tabularnewline %
& Infusion-related reaction & & 2\tabularnewline %
& Non-cardiac chest pain & & 3\tabularnewline %
& Pain & & 3\tabularnewline %
\multicolumn{4}{c}{Immune system disorders
  \cellcolor[gray]{0.9}}\tabularnewline %
& Allergic reaction & & 2\tabularnewline %
& & Anaphylaxis \tabularnewline %
\multicolumn{4}{c}{Infections and infestations
  \cellcolor[gray]{0.9}}\tabularnewline %
& Infection \footnote{Infection may include any of the 75
  infection sites under the INFECTIONS AND INFESTATIONS SOC.} & &
3\tabularnewline %
& Infections and infestations - Other (peri-rectal abscess) & &
\tabularnewline %
\multicolumn{4}{c}{Injury, poisoning and procedural complications
  \cellcolor[gray]{0.9}}\tabularnewline %
& & Gastrointestinal anastomotic leak \tabularnewline %
& Wound dehiscence & & 2 \tabularnewline
\multicolumn{4}{c}{Investigations
  \cellcolor[gray]{0.9}}\tabularnewline %
```

```
& \Gls{alt} increased & & 3\tabularnewline %
& \Gls{ast} increased & & 3\tabularnewline %
& Blood bilirubin increased & & 2\tabularnewline %
& Alkaline phosphatase increased & & 3\tabularnewline %
& Cardiac troponin I increased & \tabularnewline %
& \Gls{anc} decreased & & 3\tabularnewline %
& Weight loss & & 3\tabularnewline %
& \Gls{wcc} decreased & & 3\tabularnewline %
\multicolumn{4}{c}{Metabolism and nutrition disorders
  \cellcolor[gray]{0.9}}\tabularnewline %
& Anorexia & & 3\tabularnewline %
\multicolumn{4}{c}{Musculoskeletal and connective tissue disorders
  \cellcolor[gray]{0.9}}\tabularnewline %
& Arthralgia & & 3\tabularnewline %
& Musculoskeletal and connective tissue disorder - Other (bone
metaphyseal dysplasia)\footnote{Metaphyseal dysplasia was observed
  in young patients who still have active epiphyseal growth
  plates.}  & \tabularnewline %
& Myalgia & & 3\tabularnewline %
& Osteonecrosis of jaw \footnote{Cases of osteonecrosis of the jaw
  have been reported in cancer patients in association with
  bevacizumab treatment, the majority of whom had received prior
  or concomitant treatment with \gls{iv} bisphosphonates.} &
\tabularnewline %
\multicolumn{4}{c}{Nervous system disorders
  \cellcolor[gray]{0.9}}\tabularnewline %
& Dizziness & & 2\tabularnewline %
& Headache & & 3\tabularnewline %
& & Intracranial hemorrhage \tabularnewline %
& & Ischemia cerebrovascular \tabularnewline %
& Peripheral sensory neuropathy \footnote{ Increased rate of
  peripheral sensory neuropathy has been observed in trials
  combining bevacizumab and chemotherapy compared to chemotherapy
  alone.}& \tabularnewline %
& & \Gls{rpls} & \tabularnewline %
& Syncope & \tabularnewline %
\multicolumn{4}{c}{Renal and urinary disorders
  \cellcolor[gray]{0.9}}\tabularnewline %
& & Acute kidney injury \tabularnewline %
& Hematuria & & 3\tabularnewline %
& Proteinuria & & 2\tabularnewline %
& & Renal and urinary disorders - Other (Nephrotic Syndrome) &
\tabularnewline %
& & Urinary fistula \tabularnewline %
\multicolumn{4}{c}{Reproductive system and breast disorders
  \cellcolor[gray]{0.9}}\tabularnewline %
Reproductive system and breast disorders - Other (ovarian failure)
\footnote{Ovarian failure, defined as amenorrhea lasting 3 or more
  months with \gls{fsh} elevation ($\geq$ 30 mIU/mL), was
  increased in patients receiving adjuvant bevacizumab plus
  mFOLFOX compared to mFOLFOX alone (34\% vs. 2\%). After
  discontinuation of bevacizumab, resumption of menses and an
  \gls{fsh} level \textless 30 mIU/mL was demonstrated in 22\%
  (7/32) of these women. Long term effects of bevacizumab exposure
  on fertility are unknown.}  \tabularnewline %
& & Vaginal fistula \tabularnewline %
& Vaginal hemorrhage & & 3\tabularnewline %
\multicolumn{4}{c}{Respiratory, thoracic and mediastinal disorders
  \cellcolor[gray]{0.9}}\tabularnewline %
& Allergic rhinitis & & 3\tabularnewline %
& & Bronchopleural fistula \tabularnewline %
& & Bronchopulmonary hemorrhage \tabularnewline %
& Cough & & 3\tabularnewline %
& Dyspnea & & 2\tabularnewline %
& Epistaxis & & 3\tabularnewline %
& Hoarseness & & 3\tabularnewline %
& & Respiratory, thoracic and mediastinal disorders - Other
(nasalseptal perforation) \tabularnewline %
& & Respiratory, thoracic and mediastinal disorders - Other
(tracheo-esophageal fistula) \tabularnewline %
\multicolumn{4}{c}{Skin and subcutaneous tissue disorders
  \cellcolor[gray]{0.9}}\tabularnewline %
& Pruritis & & 2\tabularnewline %
& Rash maculo-papular & & 2\tabularnewline %
& Urticaria & & 2\tabularnewline %
\multicolumn{4}{c}{Vascular disorders
  \cellcolor[gray]{0.9}}\tabularnewline %
Hypertension & & & 3\tabularnewline %
& Thromboembolic event & & 3\tabularnewline %
& & Vascular disorders - Other (arterial thromboembolic event)
\footnote{Arterial thromboembolic event includes visceral arterial
```

```
      ischemia, peripheral arterial ischemia, heart attack, and
      stroke.}  \tabularnewline %
  \end{longtable}
\end{center}
%
The following have also been reported with bevacizumab but causality
has not been established.
%
\begin{center}
 \begin{longtable}{ @{}
      >{\raggedright}p{7cm}
      >{\raggedright}p{7cm}
      @{}
    } \toprule
    \multicolumn{2}{c}{Other adverse events reported with bevacizumab}
    \tabularnewline %
    \midrule %
    \textsc{Body system}& \textsc{Adverse event} \tabularnewline
    \midrule
    \endfirsthead
    \caption{Possible adverse events (continued)}\tabularnewline %
    \midrule %
    \textsc{Body system}& \textsc{Adverse event} \tabularnewline %
    \endhead
    \endfoot
    \bottomrule
    \endlastfoot
    Blood and lymphatic system disorders & Blood and lymphatic system
    disorders - Other (idiopathic thrombocytopenia
    purpura)\tabularnewline %
    & Disseminated intravascular coagulation\tabularnewline %
    Cardiac disorders & Pericardial effusion \tabularnewline %
    General disorders conditions & Gait disturbance \tabularnewline %
    & Sudden death (not otherwise specified)\tabularnewline %
    Hepatobiliary disorders & Hepatic failure\tabularnewline %
    Infections and infestations & Infections and infestations - Other
    (aseptic meningitis)\tabularnewline %
    Investigations & Platelet count decreased\tabularnewline %
    Metabolism and nutrition disorders & Hyponatremia\tabularnewline %
    Musculoskeletal and connective tissue disorders & Musculoskeletal
    and connective tissue disorder - Other (aseptic necrotic
    bone)\tabularnewline %
    & Musculoskeletal and connective tissue disorder - Other
    (myasthenia gravis)\tabularnewline %
    Nervous system disorders & Dysgeusia\tabularnewline %
    & Peripheral motor neuropathy \tabularnewline %
    & Seizure\tabularnewline %
    Psychiatric disorders & Confusion\tabularnewline %
    Respiratory, thoracic and mediastinal disorders & Adult
    respiratory distress syndrome\tabularnewline % &
    Pneumonitis\tabularnewline %
    & Pneumothorax\tabularnewline %
    & Pulmonary hypertension\tabularnewline %
    Skin and subcutaneous tissue disorders & Palmar-plantar
    erythrodysesthesia syndrome\tabularnewline %
    & Skin ulceration\tabularnewline %
  \end{longtable}
\end{center}

\subsubsection{Bevacizumab Dose Modifications}

There will be no dose reduction for bevacizumab. Treatment should be interrupted or stopped for
certain adverse events, as described in table \ref{tab:dm}. If bevacizumab is
interrupted for \textgreater 8 weeks, the patient
should stop bevacizumab treatment on protocol.

\begin{center}
  \label{tab:dm}
  \begin{longtable}{@{}
      >{\raggedright}p{2cm}
      >{\raggedright}p{3cm}
      p{9cm}
      @{}}
    \toprule
    \multicolumn{3}{c}{Treatment Modification for Bevacizumab-Related
      Adverse Events}\tabularnewline \hhline{---} %
    \textsc{Event}& \textsc{Grade} & \textsc{Action to be
       taken}\tabularnewline %
    \midrule %
    \endfirsthead
    \caption{Treatment modifications (continued)}\tabularnewline %
```

```latex
\midrule %
\textsc{Event}& \textsc{Grade} & \textsc{Action to be
  taken}\tabularnewline %
\midrule %
\endhead
\endfoot
\bottomrule
\endlastfoot
\multirow{3}{*}{\parbox{2cm}{\raggedright Allergic reactions or
    Acute infusional reactions/ cytokine release syndrome}} &
Grade 1-3 & If infusion-related or allergic reactions occur,
premedications should be given with the next dose and infusion
time may not be reduced for the subsequent
infusion.\tabularnewline %
& & For patients with grade 3 reactions, bevacizumab infusion
should be stopped and not restarted on the same day. At the
physicians’ discretion, bevacizumab may be permanently stopd or
re-instituted with premedications and administered patient should
be closely monitored for a duration comparable to or longer than
the duration of the previous reactions.\tabularnewline %
& Grade 4 & Stop bevacizumab\tabularnewline %
\hhline{---} %
\glsunset{cns} % show CNS as abbreviation first time
Arterial Thrombosis: Cardiac ischemia/ infarction, \gls{cns} ischemia
(TIA, CVA), any peripheral or visceral arterial ischemia/
thrombosis & Grade 2 & If new or worsened since bevacizumab
therapy: Stop bevacizumab\tabularnewline %
& Grade 3-4 & Stop bevacizumab\tabularnewline %
\hhline{---} %
Venous Thrombosis & Grade 3 or asymptomatic grade 4 & Hold
bevacizumab treatment. If the planned duration of full-dose
anticoagulation is \textless 2 weeks, bevacizumab should be held
until the full-dose anticoagulation period is
over.\tabularnewline %
& & If the planned duration of full-dose anticoagulation is
\textgreater 2 weeks, bevacizumab may be resumed during the period
of full-dose anticoagulation if \emph{all} of the criteria below are
met:\tabularnewline %
& & -- The subject must have an in-range INR (usually 2-3) on a
stable dose of warfarin or be on a stable dose of heparin prior to
restarting bevacizumab\tabularnewline %
& & -- The subject must not have pathological conditions that
carry high risk of bleeding (eg, tumor involving major vessels or
other conditions)\tabularnewline %
& & -- The subject must not have had hemorrhagic events while on
study\tabularnewline %
& & If thromboemboli worsen/recur upon resumption of study
therapy, stop bevacizumab\tabularnewline %
& Grade 4 (symptomatic) & Stop bevacizumab\tabularnewline %
\hhline{---} %
Hypertension \footnote{Definitions \begin{description}
  \item [grade 1] asymptomatic, transient (\textless 24 hours)
    increase by \textgreater 20 mmHg (diastolic) or to
    \textgreater 150/100 if previously \gls{wnl}; intervention not
    indicated
  \item [grade 2] recurrent or persistent (\textgreater 24 hours)
    or symptomatic increase by \textgreater 20 mmHg (diastolic) or
    to \textgreater 150/100 if previously \gls{wnl}; monotherapy may be
    indicated
  \item [grade 3] requiring more than one drug or more intensive
    therapy than previously
  \item [grade 4] life threatening (eg, hypertensive crisis)
  \end{description}
} & \multicolumn{2}{p{12cm}}{\myTwoCol{Treat with antihypertensive
  medication as needed. The goal of BP control should be
  consistent with general medical practice.}}\tabularnewline %
& Grade 1 & Consider increased BP monitoring\tabularnewline %
& Grade 2 asymptomatic & If diastolic BP \textless 100 mmHg: begin
anti-hypertensive therapy and continue
bevacizumab\tabularnewline %
& Grade 2-3 & If symptomatic or diastolic BP \textgreater 100
mmHg: hold bevacizumab until symptoms resolve and BP \textless
160/90mmHg\tabularnewline %
& Grade 4 & Stop bevacizumab\tabularnewline %
\hhline{---} %
Congestive Heart Failure & Grade 3-4 & Stop
bevacizumab\tabularnewline %
\hhline{---} %
Proteinuria & \multicolumn{2}{p{12cm}}{\myTwoCol{Proteinuria should be
  monitored by urine analysis for \gls{upc}
  ratio prior to every other dose of bevacizumab}}\tabularnewline %
& \gls{upc} ratio \textless 3.5 & Continue bevacizumab\tabularnewline %
```

```
    & \gls{upc} ratio $\geq$ 3.5 & Hold bevacizumab until \gls{upc} \textless
    3.5\tabularnewline %
    & Grade 4 or nephrotic syndrome & Stop
    bevacizumab\tabularnewline %
    \hhline{---} %
    Hemorrhage (\gls{cns} or pulmonary) & Grade 2-4 & Stop
    bevacizumab\tabularnewline %
    Hemorrhage (non-\gls{cns}; non-pulmonary) & Grade 3 & Patients receiving full-dose
    anticoagulation should stop bevacizumab\tabularnewline %
    & & For patients not on full-dose anticoagulation, hold
    bevacizumab until all of the following criteria are
    met:\tabularnewline %
    & & - the bleeding has resolved and Hb is stable\tabularnewline %
    & & - there is no bleeding diathesis that would increase the risk
    of therapy\tabularnewline %
    & & - there is no anatomic or pathologic condition that could
    increase the risk of hemorrhage recurrence.\tabularnewline %
    & & Patients who experience recurrence of grade 3 hemorrhage
    should stop study therapy\tabularnewline %
    & Grade 4 & Stop bevacizumab\tabularnewline %
    \hhline{---} %
    \glsunset{pres} % show PRES as abbrev first time
    \gls{rpls} or \gls{pres} & \multicolumn{2}{p{12cm}}{\myTwoCol{Hold bevacizumab if
      symptoms/signs suggestive of \gls{rpls}; subsequent management should
      include \gls{mri} scans and control of hypertension.  Stop bevacizumab upon
      diagnosis of \gls{rpls}}}\tabularnewline %
    \hhline{---} %
    \multicolumn{2}{p{5cm}}{Wound dehiscence requiring medical or
      surgical intervention} & Stop bevacizumab\tabularnewline %
    \hhline{---} %
    \multicolumn{2}{p{5cm}}{GI perforation, GI leak or fistula} & Stop
    bevacizumab\tabularnewline %
    \hhline{---} %
    Bowel obstruction & Grade 2 requiring medical intervention & Hold
    bevacizumab until complete resolution, with a minimum of 4 weeks
    after surgery.\tabularnewline %
    & Grade 3-4 & Hold bevacizumab until complete
    resolution\tabularnewline %
    & & If surgery is required, patient may restart bevacizumab after
    full recovery from surgery, and at investigator's
    discretion\tabularnewline %
    \hhline{---} %
    \multirow{2}{*}{\parbox{2cm}{\raggedright Other unspecified
        bevacizumab-related AEs (except controlled nausea/ vomiting)}}
    & Grade 3 & Hold bevacizumab until symptoms resolve to $\leq$
    grade 1\tabularnewline %
    & Grade 4 & Stop bevacizumab\tabularnewline %
    & & Upon consultation with the study chair, resumption of
    bevacizumab may be considered if a patient is benefiting from
    therapy and the grade 4 toxicity is transient, has recovered to
    $\leq$ grade 1 and unlikely to recur with
    retreatment\tabularnewline %
    \tabularnewline
    % \tabularnewline % need extra line due to long text in multirow above
  \end{longtable}
\end{center}

  \section{Monitoring}
  Patients will follow up with their Oncologist at monthly
  intervals. \gls{mri}s will be repeated every 2 months, or more frequently if
  there are new symptoms or signs concerning for disease progression. \Gls{kps}
  will be assessed at each visit.

  \section{Treatment at time of progression}
  At time of subsequent progression, the patient will be re-evaluated
  at our multidisciplinary conference. Decisions will be
  individualized but will include the options of further surgery,
  further \gls{rt}, a change in chemotherapy, treatment with
  electrical fields (NovoTTF \textregistered) and Palliative care.

\section{Statistical considerations}

\subsection{Sample size and power justification}

The primary objective of this study is to determine whether extensive
resection plus bevacizumab (experimental arm) will improve the
overall survival compared to open biopsy plus bevacizumab (control
arm). The division into experimental and control arms is somewhat
arbitrary as both are established treatements at the time of progression
and neither has clearly been shown to be superior.

Essentially, this is a Phase III design. The randomization of
```

```
experimental and control arms is $1:1$. The null hypothesis is that the median PFS for both arms will be
18 weeks, based on data from two Phase II trials of bevacizumab at
time of recurrence. \cite{Friedman2009}\cite{Kresyl2009} The
alternative hypothesis is that patients treated with \emph{an attempt
  at complete resection} plus bevacizumab will have an improvement in
overall survival to 23 weeks (\gls{hr} of 1.3). This is based on a
review of existing literature (retrospective series) on this subject,
which is detailed in the article describing this trial.

Following the methods of Therneau, we assume that the survival
distributions will be exponential. \cite{Therneau2000} The \gls{hr} is
thus the reciprocal of the ratio of median survival times. We aim for
a power of 80\% and a significance level of 0.05 (two-sided). This
would result in a sample size of 456 (assuming all patients are
treated as assigned and followed until to to progression). A typical
range of power and sample sizes is illustrated in table \ref{tab:pwr}
for the above significance level, following the method of
Schoenfeld. \cite{Schoenfeld1983}

\glsreset{hr}
\begin{table}[!h]
\caption{Sample size for $p<0.05$ (two-sided)}
\begin{center}
\label{tab:pwr}
  \begin{tabular}{@{} l l l l @{}}
    \toprule
    & \multicolumn{3}{c}{\Gls{hr}} \tabularnewline
    \cmidrule {2-4}
    Power & 1.2 & 1.4 & 2.0 \tabularnewline
    \midrule
    70\% & 742 & 218 & 51 \tabularnewline
    80\% & 945 & 277 & 65 \tabularnewline
    90\% & 1264 & 370 & 87.5 \tabularnewline
    \bottomrule
  \end{tabular}
\end{center}
\end{table}

 Given
the risks associated with overestimating the effect size in the study,
which would lead to the study being underpowered, we prefer the more
conservative margin of a \gls{hr} of 1.2. Thus, we aim for for a sample
size of 945.

Following a review of cases over the preceding 3 years (2009-2012), we estimate
that our institution sees 50-70 cases/year of first recurrence of
\gls{gbm} who had previously received standard of care (the Stupp
regimen, i.e. concurrent \gls{rt} and \gls{tmz} followed by adjuvant
\gls{tmz}). \cite{Stupp2005} This numers seen appear to be stable over
this time period. We will include those who were receiving this
 regimen as part of another trial. We estimate that 60-70\% will
be eligible for entry, and agreeable, to participation in the study,
giving approximately 40 entrants/year.

Given the more optimistic threshold of a \gls{hr} of
1.3, the trial would take 11 years to accrue. With the more
conservative \gls{hr} of 1.2, the trial would need to run
for $c.$ 23 years.

\subsection{Patient accrual}

This study is projected to accrue 3-4 patients/month. If
the average accrual is \textless 2 patients/month (6 months after trial activation), the study will be re-evaluated with respect to
feasibility.

\subsection{Patient selection and randomization}

All cases will be reviewed at our weekly weekly Multidisciplinary
Central Nervous System Tumor Conference.
Enrollment will be considered for all patients meeting the eligibility
criteria. \ref{sec:ec}
Patients will be stratified at the time of enrollment; this will be
followed by radomization, as shown in the study schema \ref{sec:sc}.

\subsection{Analysis plans}

In general, comparisons between groups will have a significance (type
I error) of $p < 0.05$ (2-sided).

Progression-free and overall survival rates will be estimated using
the Kaplan-Meier method. \cite{Kaplan1958} Differences between
treatment arms will be tested with the log-rank
test. \cite{Mantel1966} \Gls{pfs6} and \gls{mos} will be measured from the date
```

```latex
of randomization to the date of death or, otherwise, to the last
follow-up date on which the patient was known to be
alive.

Proportions/ rates will be
modelled using an exact binomial distribution, with 95\%
confidence intervals. These are: \gls{pfs6}, objective response, grade 3+
toxicities and acute or delayed \gls{cns} toxicities. For secondary
endpoints, no adjustament is planned for multiple comparisons as these are exploratory tests.

\Gls{mos} and \gls{pfs6} will also be modelled with multivariate
analyses using the Cox proportional-hazards model. The
planned covariates of interest are:

\begin{itemize}
\item Protocol treatment (biopsy or extensive resection)
\item Age
\item Tumor volume at time of recurrence
\item \gls{kps}
\item \Gls{nrg}. \\ Number of critical areas of cortex involved (0-3)\footnote{These
    are
    \begin{itemize}
    \item Motor
    \item Speech
    \item MCA i.e. referring to affected areas adjacent to the M1/M2
      areas of the Middle Cerebral Artery
    \end{itemize}}
\item Time since original diagnosis
\end{itemize}

The assumption of roportional hazards will be checked using residuals and time-varying coefficients. If the data
clearly do not follow proportional hazards, other statistical models
will be used to fit the data instead. Possible alternatives are to use
the stratified Cox proportional hazard model, accelerated failure
model, or to partition the time axis into sections where proportional
hazards assumption holds.

\subsection{Interim toxicity and futility analysis}

Interim analysis will be undertaken on an annual basis following the
methods of O'Brien and Fleming. \cite{Obrien1979} The study will be
terminated of study should these initial results indicate a marked
superiority of one treatment over the other.

\subsection{Significance Testing for Final Analysis}
The final analysis will be performed on an intention-to-treat basis, such
that all eligible cases on the study will be included in the arm to
which they were randomized, regardless of what treatment the patients
actually received. The analysis to report the final results of
treatment comparison between the experimental arm and the control arm
will be undertaken once the required sample size has been accrued. If
the $p$ value is less than protocol-specified $0.05$ (two-sided), the
study Statistician will reject the null hypothesis and conclude that
observing such a dataset would be highly unlikely if the outcome in
both arms was really similar. Thus the data would support the
hypothesis that the experimental arm improves progression-free
survival. The final report will include all information reported in
the interim analyses as well as treatment compliance and adverse events.

\section{Data collection}

\subsection{Data storage and analysis}
The Research Nurse assigned to this study will tabulate, store, and
secure data in a single password-protected Excel\textregistered
\ database located on a password-protected computer in their locked
office.  Data analysis will be completed by the Principal
Investigator and Statistician.

\subsection{Data safety monitoring plan}
The Data Safety Monitoring Plan is written to ensure the safety of the
participants and verifying the validity and integrity of the data.
All adverse events will be reported to the \gls{irb} and \gls{fda} in
an expedited manner within 10 days of occurrence. The investigator
will continue to follow or obtain documentation of the resolution
course of such an event.

\subsection{Ethical considerations and \\ protection of human subjects}
For patients that meet the inclusion criteria, informed consent will
be obtained at the time of consent for brain tumor surgery.  The
option to enter the trial will be presented to the patient, if he/she
is able to consent, or to the legal guardian/durable power of attorney
in all other circumstances.

For all patients, the risks, benefits
```

```latex
and alternatives of the trial will be discussed separately from those
associated with the planned operation. Refusal to participate in the
trial will not affect management strategies or therapeutic options.

\subsection{Potential risks}
The risks of study inclusion are similar to the risks of open
neurosurgical tumor resection.  This includes, but is not limited to,
death, coma, infection, blood loss, and permanent neurological
deficit.

\subsection{Potential benefits}
While the study provides no direct benefit to the patient, its
findings may clarify the value of greater extent of resection for
patients with recurrent glioblastoma.

\subsection{What other alternatives are there?}
If you decide not to participate in this research study, this will not
affect the care to which you are entitled and will not influence your
doctor in any way.  If you decide not to participate, your doctor will
discuss alternative treatment options with you. Some of these options
may include open surgery, open biopsy, needle biopsy, and observation.

\subsection{Protecting confidentiality}
Each consented participant will be assigned a study identification
number. All data will be collected and stored under this
identification number, with no other identifying information such as
name, date of birth, or social security number.  All collected data
will be kept on premises of the Hospital in a
password-protected desktop computer located in a locked office
accessible only to the Principal Investigator and Study Co-ordinator.
A list of corresponding medical record numbers will be similarly
tabulated and protected.

\subsection{Costs}
There will be no additional supply or equipment costs incurred to the
patient or the hospital by this study. All proposed therapies are
within the acceptable standards of current care options and will be
billed to the patients insurance company, as would otherwise be the case.

\newpage

\appendix
% turn off numbering
\setcounter{secnumdepth}{1}

\begin{center}
  {\bf APPENDIX}
\end{center}

\section{Sample consent form}

\subsection{Why am I being asked to participate in this research
  study?}

You have been identified as a possible candidate for participation in
a clinical research study called 'Biopsy vs Extensive Resection for
first recurrence of Glioblastoma: The BERG trial'. A research study
is performed when physicians try find new ways
to diagnose and/or treat illness. Since this research
study is experimental and the anticipated results have not been
proven, you need to know enough about the risks and benefits to decide
if you want to participate.  This process is called informed consent.

The Research Nurse working with the Principal
Investigator for this research study, will discuss this study with you
in detail. This Informed Consent document explains what will be
expected of you and what risks or benefits you may anticipate if you
agree to participate.  You should read this document very carefully
and ask as many questions as you need to fully understand what your
involvement in this study means. Please understand that by signing
this document you agree to participate in this experimental study.

\subsection{How many people will take part in this study?}
This Hospital is sponsoring this research
study.  It is being conducted only at this Hospital.  A target of a total of $945$ patients will participate.

\subsection{Why is this study being done?}
This research study is designed to better understand the value of
greater extent of resection when your tumor shows signs of
recurrence. By randomizing patients to extensive resection vs. open
biopsy, we will determine whether one has more clinical value than the
other. This remains an unresolved question. All proposed treatments
are already \gls{fda}-approved and
are not experimental.
```

```
\subsection{What is involved in this study?}
If you choose to participate in this study, you will be selected for
either open biopsy (where a small hole is made in the skull to take a
piece of the abnormal tissue and make a diagnosis) or microsurgical
resection (where an opening in the skull is made to expose all the
abnormal tissue and remove everything that is safe).  These are both
currently standard therapy options for patients with your diagnosis.
The purpose of the study is to determine which provides more clinical
benefit.  Following surgery, you will recover as you would normally
and then, if the tissue that is removed is diagnosed as recurrent
tumor, receive \gls{iv} bevacizumab, an anti-tumor agent that commonly given to patients at the time of recurrence. Your clinical
follow-up after this will be no different than it would be otherwise,
with routine appointments to see your Neuro-Oncologist and
Neurosurgeon at regular intervals.

\subsection{How long will I be on the study?}
If you agree to take part in this study, we will continue up with you for at least 3 years.  As stated above, no additional
appointments will be needed during this time.

\subsection{What are the risks of this study?}
The risks of this study are similar to those that you would be faced with
during routine management of your tumor.

\medskip
Risks associated with brain surgery, include death, neurological problems
such as weakness, difficulty seeing or using language, blood
loss, and infection.

\medskip
Risks associated with bevacizumab include wound infection,
hematological abnormalities (low blood counts), and gastrointestinal problems.

\subsection{Are there benefits to taking part in this study?}
This study involves treatments which would be available to you outside
the context of the study. However there may be benefits either to
having a surgical resection or to having a biopsy alone in terms of
quality of life and survival. Also, the knowledge gained from this
study may benefit others in the future.

\subsection{What other options are there?}
If you decide not to participate in this research study, this will not
affect the care to which you are entitled and will not influence your
doctor in any way. If you decide not to participate, your doctor will
discuss alternative treatment options with you. Some of these options
may include open surgery, open biopsy, needle biopsy, and observation.

\subsection{How will I be informed of additional information or new
  findings?}
If you decide to participate, your doctor will keep you informed of
any new findings that are discovered during the course of this study
which may affect your continued willingness to participate in this
study.

\subsection{What about confidentiality?}
Every attempt will be made to keep your participation in this research
study and your records confidential.  However, representatives from
the \gls{dhhs}, from the \gls{fda} and the Hospital may inspect your
research records to evaluate the results of this study.  The results of this research study may also be
published in scientific journals and/or may be presented at scientific
meetings, but your identity will not be revealed.

\medskip
A description of this clinical trial will be made available on
\href{http://www.ClinicalTrials.gov}{ClinicalTrials.gov}, as is required
by U.S. Law. This Web site will not include information that can
identify you. At most, the Web site will include a summary of the
results.  You can search this Web site at any time.

\subsection{What are the costs?}
There are no additional costs for you that are associated with this
trial. As always, you understand that you will be responsible for
payment of any bills that your insurance may refuse to pay due to your
participation in this research study.

\subsection{What happens if I am injured while participating in the study?}
If you experience an injury or adverse event, please contact the
Principal Investigator at 123 456 78910.  If the investigator determines that the injury
or adverse event is due to your participation in this research, this
will be treated, although you are always responsible for the financial
costs associated with your care. This does not waive your
rights in the event of negligence.

\subsection{What are my rights as a participant?}
```

```latex
You understand that your participation in this research study is
voluntary and that you are free to withdraw from this study at any
time.  If you decide not to participate, your doctor will not hold it
against you in any way.  If you decide to withdraw from this study at
a later date, please notify the Principal Investigator of your decision and to
discuss your alternative treatment options.

\medskip
You understand that your doctor may also stop your participation on
this study if it is felt to be in the best interest of your health.
This may be done without your consent, but alternative treatment
options will be discussed with you.

\subsection{Whom do I call if I have questions?}
If you have any questions about this research study or your
participation in it, please feel free to ask them now. If you think of
questions later or have concerns about your participation, please call
the Principal Investigator at 123 456 78910.

\medskip
If you have any questions about your rights as a research subject or
about the Institutional Review Board for Human Research (IRB) at
the Hospital. The board has reviewed this Informed
Consent document for compliance with federal guidelines. They may be
contacted at:
\bigskip
\noindent
Institutional Review Board\\
Hospital\\
Address\\
Phone no.
\bigskip
You are voluntarily deciding whether or not to participate in the
research study described in this consent form.  Your signature below
indicates that you have read and understand the information provided,
and that you have decided to participate.  You will receive a copy of
the signed informed consent document.

\subsection{Patient/ representative signature}

\bigskip \bigskip \bigskip
\noindent
\underline{\hspace{0.75\textwidth}}\\
Printed name
\newline \newline \newline \newline
\noindent
\underline{\hspace{0.75\textwidth}}\\
Signature
\newline \newline \newline
\noindent
\underline{\hspace{0.3\textwidth}}\\
Date
\newline \newline \newline \newline \newline
\noindent
\underline{\hspace{0.75\textwidth}}\\
Signature of Legally Authorized Representative
\newline \newline \newline
\noindent
\underline{\hspace{0.75\textwidth}}\\
Relationship to patient
\newline \newline \newline
\noindent
\underline{\hspace{0.75\textwidth}}\\
Reason patient is unable to sign
\newline \newline \newline
\noindent
\underline{\hspace{0.75\textwidth}}\\
Date
\newline \newline \newline
\noindent
\underline{\hspace{0.75\textwidth}}\\
Signature of Interpreter (If applicable)
%\newline \newline
\subsection{Principal investigator's signature}
\bigskip \bigskip
\noindent
\underline{\hspace{0.75\textwidth}}\\
Printed name
\newline \newline \newline \newline
\noindent
\underline{\hspace{0.75\textwidth}}\\
Signature
\newline \newline \newline
```

```latex
\noindent
\underline{\hspace{0.3\textwidth}}\\
Date

\newpage

\section{Rights of humans subjects in medical experiments}

\medskip
Any person who is requested to consent to participate as a subject in
a research study involving a medical experiment or who is requested to
consent on behalf of another has the right to:

\begin{enumerate}
\item Be informed of the nature and purpose of the experiment.
\item Be given an explanation of the procedures to be followed in the
  medical experiment, and any drug or device to be utilized
\item Be given a description of any attendant discomforts and risks
  reasonably to be expected from the experiment
\item Be given an explanation of any benefits to the subject
  reasonable to be expected from the experiment, if applicable
\item Be given a disclosure of any appropriate alternative procedures,
  drugs or devices that might be advantageous to the subject, and
  their relative risks and benefits
\item Be informed of the avenues of medical treatment, if any
  available to the subject after the experiment if complications
  should arise
\item Be given an opportunity to ask any questions concerning the
  experiment or the procedure involved
\item Be instructed that consent to participate in the medical
  experiment may be withdrawn at any time and the subject may
  discontinue participation in the medical experiment without
  prejudice
\item Be given a copy of any signed and dated written consent form
  used in relation to the experiment
\item Be given the opportunity to decide to consent or not to consent
  to a medical experiment without the intervention of any element of
  force, fraud, deceit, duress, coercion or undue influence on the
  subjects decision
\end{enumerate}

I attest that I, or my representative, discussed this study with the
above named participant or legal representative.  This person had
enough time to consider this information, had an opportunity to ask
questions, and voluntarily agreed to participate in this study.

\newpage

\section{Karnofsky performance scale}

\begin{itemize}
\item [100] Normal; no complaints; no evidence of disease
\item [90] Able to carry on normal activity; minor signs or symptoms
  of disease
\item [80] Normal activity with effort; some sign or symptoms of
  disease
\item [70] Cares for self; unable to carry on normal activity or do
  active work
\item [60] Requires occasional assistance, but is able to care for
  most personal needs
\item [50] Requires considerable assistance and frequent medical care
\item [40] Disabled; requires special care and assistance
\item [30] Severely disabled; hospitalization is indicated, although
  death not imminent
\item [20] Very sick; hospitalization necessary; active support
  treatment is necessary
\item [10] Moribund; fatal processes progressing rapidly
\item [0] Dead
\end{itemize}

\begin{thebibliography}{99}

\bibitem{Louis07} Louis DN, Ohgaki H, Wiestler OD, et al. The 2007
  WHO classification of tumours of the central nervous system. Acta
  neuropathologica 2007, 114:97-109.

\bibitem{CBTRUS2012} CBTRUS Statistical Report: Primary Brain and
  Central Nervous System Tumors Diagnosed in the United States in
  2004-2008, 2012.

\bibitem{Taal2008} Taal W, Brandsma D, de Bruin HG, et al. Incidence
  of early pseudo-progression in a cohort of malignant glioma patients
  treated with chemoirradiation with temozolomide. Cancer 2008,
```

```
  113:405-410.

\bibitem{Stupp2005} Stupp R, Mason WP, van den Bent MJ, et al.
  Radiotherapy plus concomitant and adjuvant temozolomide for
  glioblastoma. NEJM 2005, 352:987-996.

\bibitem{Wong1999} Wong ET, Hess KR, Gleason MJ, et al. Outcomes and
  prognostic factors in recurrent glioma patients enrolled onto Phase
  II clinical trials. J Clin Oncol 1999, 17:2572-2578.

\bibitem{Easaw2011} Easaw JC, Mason WP, Perry J, et al. Canadian
  Glioblastoma Recommendations Committee. Canadian recommendations for
  the treatment of recurrent or progressive glioblastoma
  multiforme. Curr Oncol 2011, 18(3):126-136.

\bibitem{NCCN2013} NCCN Guidelines Version 2.2013. Anaplastic gliomas/
  glioblastoma. \href{http://www.nccn.org/professionals/physician_gls/pdf/cns.pdf]}{Available
    online}

\bibitem{Brandes2013} Brandes AA, Bartolotti M, Franceschi E. Second
  surgery for recurrent glioblastoma: advantages and pitfalls. Expert
  Rev Anticancer Ther 2013 13(5):583-587.

\bibitem{Friedman2009} Friedman HS, Prados MD, Wen PY et al.
  Bevacizumab alone and in combination with irinotecan in recurrent
  glioblastoma. J Clin Oncol, 2009 27(28):4733-4740.

\bibitem{Mantel1966} Mantel N. Evaluation of survival data and two new
  rank order statistics arising in its consideration. Cancer Chem Rep
  1966 5:163-170.

\bibitem{Kaplan1958} Kaplan EL, Meier P. Nonparametric estimation from
  incomplete observations. J Am Stat Assoc 1958 53:457-481

\bibitem{Kresyl2009} Kreisl TN, Kim L, Moore K et al. Phase II trial
  of single-agent bevacizumab followed by bevacizumab plus irinotecan
  at tumor progression in recurrent glioblastoma. J Clin Oncol, 2009
  27(5):740-745.

\bibitem{Schoenfeld1983} Schoenfeld D. Sample-size formula for the
  proportional-hazards regression model. Biometrics 1983, 39:499-503.

\bibitem{rtog0825} Gilbert M. Phase III Double-Blind
  Placebo-Controlled Trial of Conventional Concurrent Chemoradiation
  and Adjuvant Temozolomide Plus Bevacizumab versus Conventional
  Concurrent Chemoradiation and Adjuvant Temozolomide in Patients with
  Newly Diagnosed Glioblastoma. \href{http://www.rtog.org/ClinicalTrials/ProtocolTable/StudyDetails.aspx?study=0825}{Available
    online}

\bibitem{Therneau2000} Therneau T, Grambsch P. Modeling Survival Data:
  Extending the Cox Model. Springer: New York; 2000.

\bibitem{Obrien1979} Obrien P, Fleming T. A Multiple Testing Procedure
  for Clinical Trials. Biometrics 1979, 35:549-556.

\end{thebibliography}

\end{document}
```
